# Bouts of rest and physical activity in C57BL/6J mice

**DOI:** 10.1371/journal.pone.0280416

**Published:** 2023-06-26

**Authors:** Karin Pernold, Eric Rullman, Brun Ulfhake

**Affiliations:** Division of Clinical Physiology, Department of Laboratory Medicine, Karolinska Institutet, Stockholm, Sweden; Belgrade University Faculty of Medicine, SERBIA

## Abstract

The objective was to exploit the raw data output from a scalable home cage (type IIL IVC) monitoring (HCM) system (DVC®), to characterize pattern of undisrupted rest and physical activity (PA) of C57BL/6J mice. The system’s tracking algorithm show that mice in isolation spend 67% of the time in bouts of long rest (≥40s). Sixteen percent is physical activity (PA), split between local movements (6%) and locomotion (10%). Decomposition revealed that a day contains ˜7100 discrete bouts of short and long rest, local and locomotor movements. Mice travel ˜330m per day, mainly during the dark hours, while travelling speed is similar through the light-dark cycle. Locomotor bouts are usually <0.2m and <1% are >1m. Tracking revealed also fits of abnormal behaviour. The starting positions of the bouts showed no preference for the rear over the front of the cage floor, while there was a strong bias for the peripheral (75%) over the central floor area. The composition of bouts has a characteristic circadian pattern, however, intrusive husbandry routines increased bout fragmentation by ˜40%. Extracting electrode activations density (EAD) from the raw data yielded results close to those obtained with the tracking algorithm, with 81% of time in rest (<1 EAD s^-1^) and 19% in PA. Periods ≥40 s of file when no movement occurs and there is no EAD may correspond to periods of sleep (˜59% of file time). We confirm that EAD correlates closely with movement distance (r_s_>0.95) and the data agreed in ˜97% of the file time. Thus, albeit EAD being less informative it may serve as a proxy for PA and rest, enabling monitoring group housed mice. The data show that increasing density from one female to two males, and further to three male or female mice had the same effect size on EAD (˜2). In contrast, the EAD deviated significantly from this stepwise increase with 4 mice per cage, suggesting a crowdedness stress inducing sex specific adaptations. We conclude that informative metrics on rest and PA can be automatically extracted from the raw data flow in near-real time (< 1 hrs). As discussed, these metrics relay useful longitudinal information to those that use or care for the animals.

## Introduction

In every-day life, metrics of rest and physical activity (PA) are important measures of well-being and health in humans and animals alike. These metrics are useful to monitor changes through life, impact of lifestyle, disease signature and progression, and responses to environmental conditions. Traditionally, behaviours of laboratory mice have been assessed by snapshots of home cage behaviours or out-of-cage (and everyday life context) testing, these approaches may not substitute well for cumulative unsupervised monitoring of spontaneous behaviours and their alterations over time. Efforts to solve these shortcomings dates more than a century back [1], however, due to technical obstacles it was not until the late 20^th^ century that such systems evolved (e.g. [2, 3]) and later became commercially available (e.g. Intellicage by TSE, Metris by Labora, ActiMot by TSE, and Pheno typer by Noldus; see e.g. [4–8]). Still, these systems are lab-bench type of equipment and not possible to integrate in standard holding systems of the [laboratory animals’] vivarium. More recently novel and scalable HCM systems using different monitoring techniques [9, 10] have been developed for automated non-intrusive 24/7 cumulative monitoring of home-cage activity (home-cage monitoring, HCM) e.g. [11, 12] suitable for a vivarium of small rodents. As recently reviewed [13–15], such HCM systems provide an excellent opportunity to collect cumulative unsupervised records of in-cage rest and PA on a large scale.

The purpose of this study was to characterize spontaneous in-cage activity and rest across the circadian cycle and cycles of recurrent husbandry routines over multiple weeks to provide base line data on duration and frequency of bouts of rest and PA, and how the animals use the cage floor as well as rhythmicity of rest and PA. For this purpose, we recorded cumulative data with a home-cage monitoring system (DVC® Tecniplast SpA) of C57BL/6J mice in standard IVC cages (GM500), kept either in isolation (x1) or in groups at different densities (x2, x3, x4). The DVC® system is based on twelve planar capacitance sensing electrodes situated outside and beneath the cage in a standard cage rack. The electrode array defines the spatial resolution and electrode samples are collected at 4 Hz [12, 15, 16]. The 24/7 flow of electrode reads (raw data) is processed to provide spatial and temporal information on electrode activations which is used to delineate bouts of in-cage PA and rest. With animals kept in isolation the data can be used to track the animals’ position and to monitor the animals’ in-cage movements. These capacities of the system have previously been validated towards CCD recordings [16].

In this report we present data on frequency and duration of bouts of rest and PA, across the cage floor, circadian cycle and across weeks of observation. PA bouts are split into bouts of movements on the spot (MOTS) [17, 18] and bouts of locomotion based on distance made during the bout [17, 19]. Bouts of rest were divided into short and long (≥40s) because long bouts of rest correlates closely with sleep [20–24]. Furthermore, we used electrode activation density (EAD) [12, 16] do assess the impact on in-cage synchronized-rest and PA of male and female mice housed at different densities (x1, x2, x3, and x4).

With standard desk top computers, the metrics presented herein can be extracted 24/7 in near-real time (≤ 1h) for all cages in a 60-slot IVC rack. Since mice mainly are used in experimental work as models for human conditions, this set of information about in-cage life may not only be of value to the those that care or use the mice but may prove to provide useful biomarkers that translate well to corresponding assessments made in humans.

## Materials and methods

### Mouse strain, sex, and age

Cohorts of specific pathogen free (SPF, according to FELASAs exclusion list [25, 26]) male (m) and female (f) C57BL/6J mice were delivered by car from Charles River, Germany, (Table 1). Upon arrival subjected to a brief health check, at 6–8 weeks of age mice were randomly allotted to cages and either grouped 2 (x2), 3 (x3), 4 (x4) per cage or kept in isolation (x1).

**Table 1:** 

Cohort-name	Strain & Origin	Sex	Housing density (cage #) and number of animals.	Age (in study)	Cage change intervals	Feed and drink (ad libitum)	Bedding material. Light and dark regime.	Cage enrichment
Fx4 (KI2018)	C57BL/6J Charles River, Germany	Female	4 (N = 10) Total: 40	42–129 days (˜6-19wks)	Bi-weekly incl. BW	SDS 3RM. Weakly chlorinated tap water.	100g Aspen (5x5x1mm, Tapvei). LON: 4–16 CEST.	Sizzle nest (BEDRNEST 70mm, 8g, Datesand)
Mx4 (KI2018)	C57BL/6J Charles River, Germany	Male	4 (N = 10) Total: 40	42–129 days (˜6-19wks)	Bi-weekly incl. BW	SDS 3RM. Weakly chlorinated tap water.	100g Aspen (5x5x1mm, Tapvei). LON: 4–16 CEST.	Sizzle nest (BEDRNEST 70mm, 8g, Datesand)
Fx3 (SU2020)	C57BL/6J Charles River, Germany	Female	3 (N = 10) Total: 30	38–129 days (˜6-19wks)	Bi-weekly incl. BW	1324 P IRR, Altromin. Weakly chlorinated tap water.	100g Aspen (2x2x1mm, Tapvei). LON: 9–21 CEST.	Sizzle nest (BEDRNEST 70mm, 8g, Datesand), red mouse-house (Tecniplast).
Mx3 (SU2020)	C57BL/6J Charles River, Germany	Male	3 (N = 10) Total: 30	38–129 days (˜6-19wks)	Bi-weekly incl. BW	1324 P IRR, Altromin. Weakly chlorinated tap water.	100g Aspen (2x2x1mm, Tapvei). LON: 9–21 CEST.	Sizzle nest (BEDRNEST 70mm, 8g, Datesand), red mouse-house (Tecniplast).
Mx2 (KI2019)	C57BL/6J Charles River, Germany	Male	2(N = 10) Total: 20	97–124 days (˜14-18wks)	Bi-weekly incl. BW	SDS 3RM. Weakly chlorinated tap water.	100g Aspen (5x5x1mm, Tapvei). LON: 4–16 CEST.	Sizzle nest (BEDRNEST 70mm, 8g, Datesand)
Fx1 (SU2021)	C57BL/6J Charles River, Germany	Female	1 (N = 10) Total: 10	58–100 days (˜8-14wks)	Bi-weekly incl. BW	1324 P IRR, Altromin. Weakly chlorinated tap water.	100g Aspen (2x2x1mm, Tapvei). LON: 9–21 CEST.	Sizzle nest (BEDRNEST 70mm, 8g, Datesand), red mouse-house (Tecniplast).

### Holding and husbandry conditions

**Fig 1:**
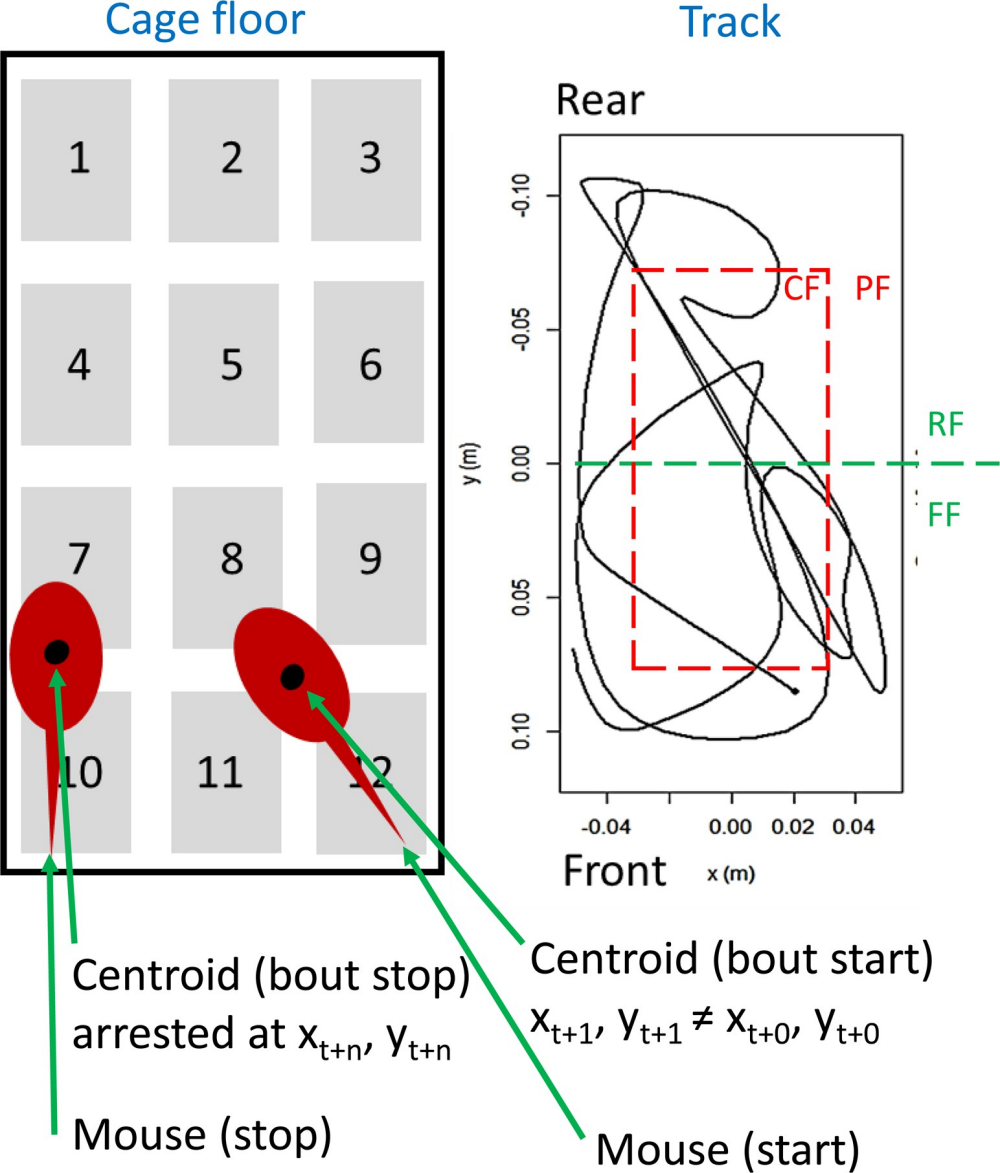
An electrode capacitance is calculated from the actual reading of the electrode at t and the base-line value. The values from the 12 electrodes are then used to calculate the x, y position of the mouse centroid (left panel). A PA bout is initiated when x, y changes in successive samples and continuous until the mouse is still again (no change in x, y between successive samples). The time-series of x, y coordinates during a movement bout is used to plot the bout trajectory (right panel) and to decide if it is a MOTS or locomotor bout. Inserted into the track diagram (right panel) is how the cage floor was divided into a front(FF)-rear(RF) area (green line and text) and a central(CF)-peripheral(PF) area of equal size (red line and text), respectively. For further information see text.

Mice were kept in individually ventilated caged (IVC) of type GM500 (Tecniplast SpA, Italy) in a DVC system (Tecniplast SpA) (Table 1). IVC cages are ventilated with 75 HEPA14 filtered air exchanges per hour, the air is taken from the holding room and let out through a separate outlet. The holding room has a 12-12h dark/light cycle (DL; Zeitgeber time (ZT) 0–12 (L) and 12-24(D)) with white light level at 15–40 Lux inside the cage. Cohort Fx1, Fx3 and Mx3 had had lights on/ lights off with dawn and dusk of 60 min (See Fig 1 in S1 File), while cohorts Mx2, Mx4 and Fx4 had sudden change of the lightning conditions in the vivarium.

All cages had 100g aspen chips 2 or 5 mm (Tapvei, Finland) as bedding, nestlets, Bed-r’Nest or sizzle nest and several (Fx3, Mx3, and Fx1; see Table 1) of the cohorts also had a red polycarbonate mouse house (Tecniplast SpA) as enrichment. The husbandry routines included cage change every other week (see Table 1; (whole cage was changed but red house and some of the soiled beddings were moved along with the animals to the new cage) and also body weighing (Table 1).

Handling of the mice by staff was either by using cupped hand or by forceps at the tail root, all mice in the different groups were subjected to both handling routines. The holding units were subject to health inventories according to FELASA’s recommendation for a sentinel reporting system (i.e. the subjects of the study were not directly affected by the health inventory) four times a year [25, 26] and during the study period the output from the sentinel system met the FELASA exclusion list for specific pathogen free animals (SPF). Surveillance of health and welfare included daily check-ups and individual examination during the cage-change and weighing every other week. Health is assessed according to a scoring list deployed at all facilities on Karolinska Institutet and Stockholm University, amended by special requirements stated in the ethic permit. When we weighed the animals, these metrics along with the scoring list formed the basis of the welfare and health check-ups. As needed the designated veterinarian of the facility was consulted.

### Ethical considerations

Both husbandry routines and applied procedures followed applicable guidelines and were agreed upon, reviewed, and approved by the Regional Ethics Council, Stockholms Regionala Djurförsöksetiska nämnd; project licenses N116-15, N184/15 plus amendments and project license 9467–2020 with addendum 12337–2021. No special requirements for health and welfare checks beyond those already implemented at the facility (see above) were required by the permits. DVC records of animals kept in isolation derived from 10 cages serving as control animals for an unrelated experiment granted in permit 9467–2020 with addendum 12337–2021.

### DVC recordings

In total, recordings were collected from 60 cages arranged by sex and housing density in 6 cohorts (Table 1) maintained at the Wallenberg Laboratory on Karolinska Institutet or ECF at Stockholm University both in Stockholm, Sweden. Here we have collected new but have also re-analysed previous [27] cumulative DVC recordings for the purpose of revealing patterns of rest and activity. In doing so we reduced number of animals needed for the study.

The core of the DVC system is an electronic sensor board installed externally and below each standard IVC cage of a rack. The sensor board is composed of an array of 12 electrodes and employs a capacitive-based sensing technology (CST). A proximity sensor measures the electrical capacitance of each of the 12 electrodes at 4 Hz (i.e., every 250 msec). The electrical capacitance is influenced by the dielectric properties of matter close to the electrode, leading to measurable capacitance changes due to the presence/ movement of animals in the cage above. Thus, movements across the electrode array are detected and recorded as alterations in capacitance [12, 16].

### REM unit

At the ECF facility, the DVC rack was equipped with a REM unit (Tecniplast SpA) which record 24/7 noise (audible range), vibrations (acceleration), light level (Lux) and presence of humans in front of the rack. Temperature and humidity were regulated by the Scanclime airflow unit modulating these parameters of the air in-flow. At the Wallenberg facility, the air-flow unit uses the air of the holding room, and records of temperature and humidity are those of the holding room. Light level in front of the cages across the DL cycle applicable to cohorts x1 and x3 is shown in Fig 1 in S1 File.

### Tracking rest and movements of single housed female mice

In this study we used two different analytical approaches namely tracking and electrode activation density (EAD; see below) based on the CST to reveal pattern of rest and activity in single housed animals. For detailed description of these metrics we refer to the manufacturer (tracking) and previous publications (EAD; [12, 16]. Both metrics have been validated towards video tracking [16]. Briefly, and as described by the manufacturer the mouse position on the cage floor is determined by estimating a short-period baseline *R*_k_(t) per each electrode *k* and per each time *t* as the maximum capacitance measurement within a 1-minute moving window. *d*_k_(*t*) is the difference between the estimated baseline *R*_*k*_(*t*) and the current capacitance measurement *c*_*k*_(*t*) of each electrode. The mouse position is determined as the centroid of the coordinates of the 12 electrodes weighed by their corresponding signal drop *d*_*k*_(*t*) with a resolution of ˜1mm [15, 16]. A Gaussian filter is applied across time and space to smooth the trajectory.

The tracking algorithm was used to differentiates between the mouse being still (resting), i.e. no change in *x*, *y* position of the centroid between successive samples, and in motion where *x*, *y* position change between successive samples (Fig 1). For practical reasons, cut off point for motion was set when the difference in successive samples of *x*, *y* [in Euclidian distance] ≥ 1 mm between sample. Based on previous records of step length for this strain and sex, motion-episodes were divided into local movement (movement-on-the-spot, MOTS) [17] being less than one average stride length (65 mm) in radial distance from the starting point and locomotion when the trajectory covered at least one full stride length [19]. Tracking of individual animals is possible only when animals are kept in isolation and were thus executed on data records from cohort Fx1 only (Table 1).

The tracking records covering 6 weeks for each cage, are time-series with bouts of motion (locomotion and MOTS) interrupted by bouts where the animal was still. The duration and frequency of bouts (still, MOTS and locomotion) were calculated, including distance (m) and speed (ms^-1^) along with other metrics when the animal moved. Bouts of being still were subdivided into short and long bouts of rest, based on bout duration. Earlier studies of mice and rats have shown that periods of rest lasting 40s or longer correlate closely with sleep [20–23, 28]. This was used as cut point between long and short rest. The composition of bouts for each cage was compared as aggregated values, light vs dark (night time) hrs., circadian cycle and days, and across weeks, including impact by husbandry routines [12, 16, 27].

### Rest and physical activity (PA) derived from EAD of single-housed female mice

An alternative to tracking is to extract the spatial and temporal pattern of CST activations as previously described in [12, 16]. In cooperation with the DVC team at Tecniplast, CST activations was extracted from the raw data by taking the average of two consecutive capacitance readings and calculate the difference with the average of the following two readings (two windows, W2) and compared the absolute value of the capacitance change to the lowest possible threshold (λ) that did not pass-through noise generated in empty cages as activity (λ = 1.25). The output is binary from the comparison of successive samples, either an electrode is activated (1) or not (0). The method has previously been validated against CCD-tracking [16].

The output was averaged s^-1^ and referred to as electrode activation density s^-1^ (EAD). The average read s^-1^ (average global activation) from all 12 electrodes have been used in most of the analyses of this study. In addition, the density of activations in the front (electrodes 7 to 12) and rear (electrodes 1–6) of the cage was compared to assess polarity of rest and activity, and as a proxy for the spatial extent of PA we computed the number of unique electrodes activated s^-1^ (UnEA).

**Fig 2:**
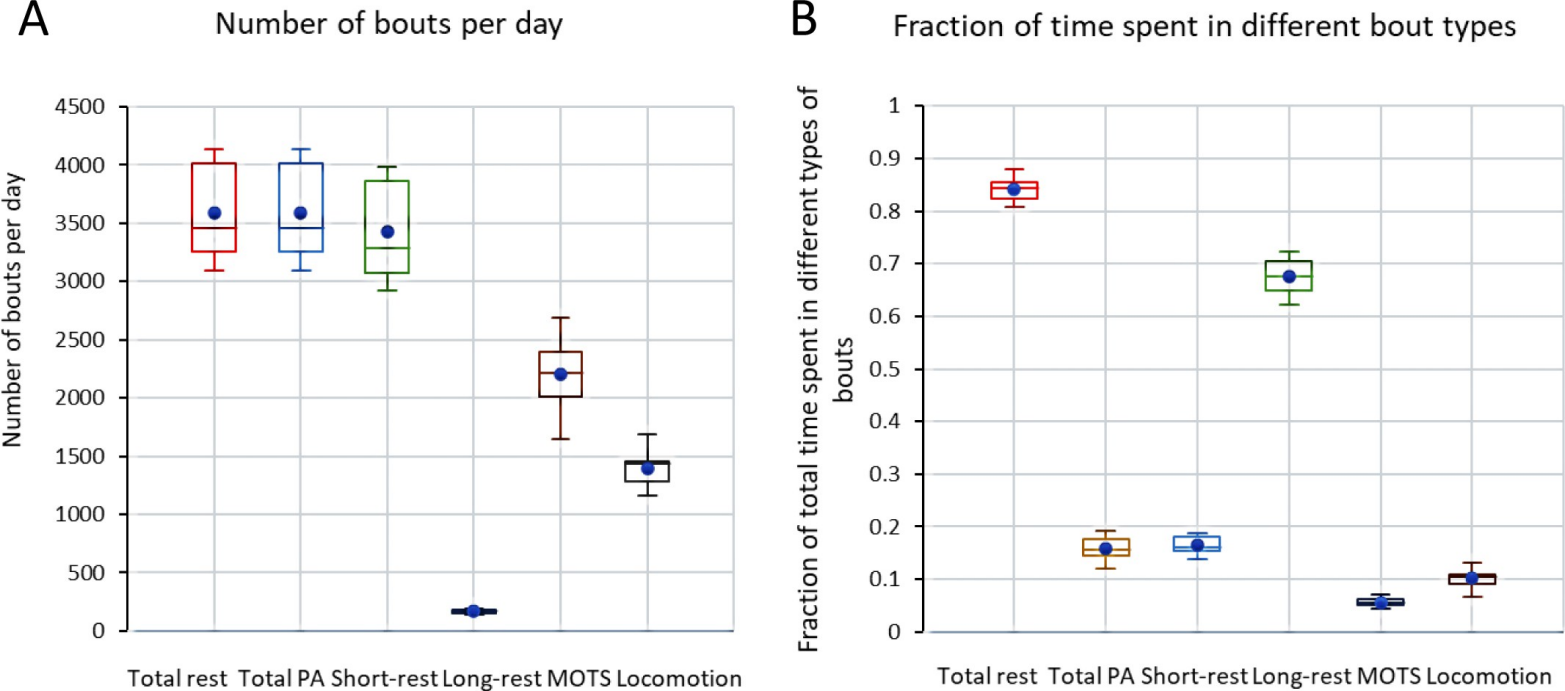
A Boxplots showing number of PA and rest bouts per day for single housed female mice. Rest is further divided into short and long rest, and PA in local movements (MOTS) and locomotor bouts. B Boxplots showing time spent in rest, long rest, short rest, and PA, in MOTS, and locomotion as fraction of total time. Values indicated are mean, 1st and 3rd quartiles and range. Mean value has been indicated with a blue circle.

Having both the tracking (still-movement) and the EAD (still-PA) records from the animals kept in isolation, allowed us to assess to what extent these two metrics co-variates. The tracking and EAD files for each animal were aligned using the time stamps and compared. Our data confirms previous observations of a close correlation between EAD and tracking distance per hour [16] (See Fig 2 in S1 File) and the correlation is very close (˜r_s_>0.95; *idem*). The correlations between distance made during a locomotor or a MOTS bout, on the one hand, and EAD per bout, on the other, revealed that the relationship between made distance and EAD was different between MOTS and locomotor bouts (See Fig 4 in S1 File) reflecting the different contents of these two bout types. The correlation between locomotor bout distance and EAD was still significant in each animal, however, less close than the cumulative distance per unit time vs. sum EAD per unit time (Fig 4 in S1 File). Although the covariation between the EAD and tracking metrics appears to be solid also over an extended period of time (c.f. [16]) and both metrics indicates that animals kept in isolation are at rest on average 81%-84% of the time (see below), there remain some discrepancies when the two data files are compared (Table 2). During 3% of the file time, the tracking coordinates do not change but EADs are recorded (˜29% of all EADs in file across 6 weeks; Table 2). Thus, there is a close but not perfect match between the two metrics.

**Table 2:** 

Activity and rest assessed by EAD (s^-1^)					
% of total time of file	Mean	SD	Median	Range	
				Min	Max
% file time at rest (act <1 s^-1^)	80.9	2.5	81.3	77.2	83.9
% of time at rest (act<1 s^-1^) and bout distance <1mm per sample	78.7	2.87	79.3	74.7	81.6
% of file time in activity (act ≥1 sec^-1^)	19.1	2.5	18.7	16.1	22.8
% of activations in file when the animal is still (< 1 mm per sample)	28.8	4.89	28.5	22.9	38.3
% of activations in file when the animal moves (≥1 mm per sample)	71.2	4.89	71.5	61.7	77.1

### Synchronized rest and group PA derived from time-series of EAD of group housed female and male mice

With group housed animals rest and PA assessed by EAD does not apply to individual animals only to the group. Rest assessed by EAD is there for referred to as synchronized rest and, moreover, PA cannot be divided into MOTS and locomotion. The raw data files were processed as for the single housed mice, the only difference being that no tracking data was available to align with due to the group housing. Furthermore, periods of rest and long rest (≥ 40s) are episodes in the time series when this was synchronized within the group of mice.

### Data processing

Data were processed through scripts in R (version 4.0.3). The following libraries were used and are hereby acknowledged: broom (ver 0.7.4), compositions (ver 2.0–0), CRamisc (ver 0.5.0.9001), data.table (ver 1.13.6), DescTools (ver 0.99.39), dplyr (ver 1.0.2), filesstrings (ver 3.2.2), ggplot2 (ver 3.3.3), gvlma (ver 1.0.0.3), labelled (ver 2.7.0), microbenchmark (ver 1.4–7), nparLD (ver 2.1), purr (ver 0.3.4), RColorBrewer (ver 1.1–2), readr (ver 1.4.0), stringr (ver 1.4.0), tibble (ver 3.0.4), tidyr (ver 1.1.2), tidyverse (ver 1.3.0), trajar package (ver 1.4.0), zoo (ver 1.8–8), Mixed Effects Models (nlme) library version 3.2.152 on R version 3.5.3.

Time-series of motion and rest generated by the tracking algorithms (Fx1 cohort only, n = 10) and time series based on EAD of rest and PA from each cage (all cages, n = 60; Table 1) were used to analyse the distribution of activity and rest by sex and housing density. In animals kept isolated, movement and rest were analysed as aggregated values or segmented into bouts as described above. The data set was then compared to the data set of EAD, i.e. activations and rest [of the same cage], matching the time-series by the time-stamps (s^-1^) to reveal the extent of covariation of metrics among isolated animals (see also [16]). While the locomotion files were divided into bouts of rest and locomotion, and further subdivided into MOTS and locomotion (see above) as well as short and long rest, respectively; the EAD time series files were divided into rest when no electrode was activated (<0.02 average activations s^-1^; i.e., <1 electrode activation s^-1^) and PA if ≥1 electrode activation(s) was recorded (≥0.02 activations s^-1^). As with the tracking time-series, episodes (bouts) of activations are intervened by episodes (bouts) of rest. Rest episodes were further subdivided into bouts of short and long rest (≥40s; see above).

Frequency and duration of bouts were saved along with average and cumulative PA during a bout. The records were used to analyse bout duration and frequency in relation to established rhythmicities [of in-cage behaviours] e.g. day vs. night, circadian, and recurring husbandry routines [10, 12, 29–34].

### Statistical analyses

Aggregated data with a normal distribution (Kolmogorov-Smirnoff test of normality) were analysed by linear-regression, or ANOVA, or mixed model ANOVA including post hoc testing. Paired and unpaired samples with a normal distribution were tested with two-sided t-test (equal or unequal variance). Effect size for variables having a normal distribution have been indicated by Hedge’s *q*, or as the coefficient of variation (r^2^) [35]. For parametric statistics we used either R scripts or the plug-in XLSTAT module running on MS Excel.

However, several metrics showed large deviations from a normal distribution and could not be normalized by Box-Cox transformation. We therefore choose to test differences across cages, housing densities and/or sexes, cage-change cycles, and days, and across weeks, by nonparametric repeated measures analysis, using the rank-based analysis of variance-type statistic (ATS), as implemented in the nparLD R Software package [36, 37]. Cages are subjects; housing density, time, event, and observation-order are within-subject factors (“sub-plot” repeated factors), and sex and housing density are between-subject factors (“whole-plot” factor) in the models used. The statistical analysis of time-series with nparLD is based on rank-order of the observed data, with the relative effect (*p*_s_) as effect size measure [37]. The difference towards parametric tests being that the rank-order not the mean difference between observations, is used to assess the probability that two sets of observations differ (*p*_s_ = 0.5 means that there is no difference in rank-order), and/or if the relative effect size varies across the time-series within the sets of observations. Comparison of two independent samples, and paired samples, were conducted using nonparametric Mann-Withney U statistics (U-test and Wilcoxon’s test for matched pairs). In these instances, we used the common language (CL) effect size statistics [38–41]. The CL effect size is based on the rank-order (rank sum) of the observed values and indicates the relative frequency with which the rank sum from one set of observations will be larger than the rank sum of a second set of observations. Correlation between metrics with unknown or a non-normal distribution was done using the nonparametric Spearman rank correlation. The Spearman correlation coefficient (rho, r_s_) indicates the effect size that range from a perfect inverse covariation (r_s_ = -1), through no covariation (r_s_ = 0) to a perfect positive covariation (r_s_ = 1) of the ranks for two parameters.

We used R scripts or XLSTAT to run the nonparametric statistical tests. Box plots indicate median, 25%-75% quartiles with max and min as bars. In addition, circular symbols indicate mean values.

## Results

### Movements and rest of single-housed female mice (Fx1)

#### Tracking of rest and movements in the home-cage

For single housed female mice (Fx1, n = 10) the total file time of 6 weeks was decomposed into bouts of rest and PA. PA bouts were further segmented in locomotor and MOTS bouts (see Material and methods and [17, 18]). During the observation period, female mice spent 84% of the time at rest with rather small difference between animals (Fig 2). Sixty-seven percent of the file time were bouts of rest lasting at least 40s. The remaining ˜16% of the time, the animals were engaged in PA split between MOTS (˜6%) and bouts of locomotion (˜10%) (*idem*).

#### Density and duration of bouts

**Fig 3:**
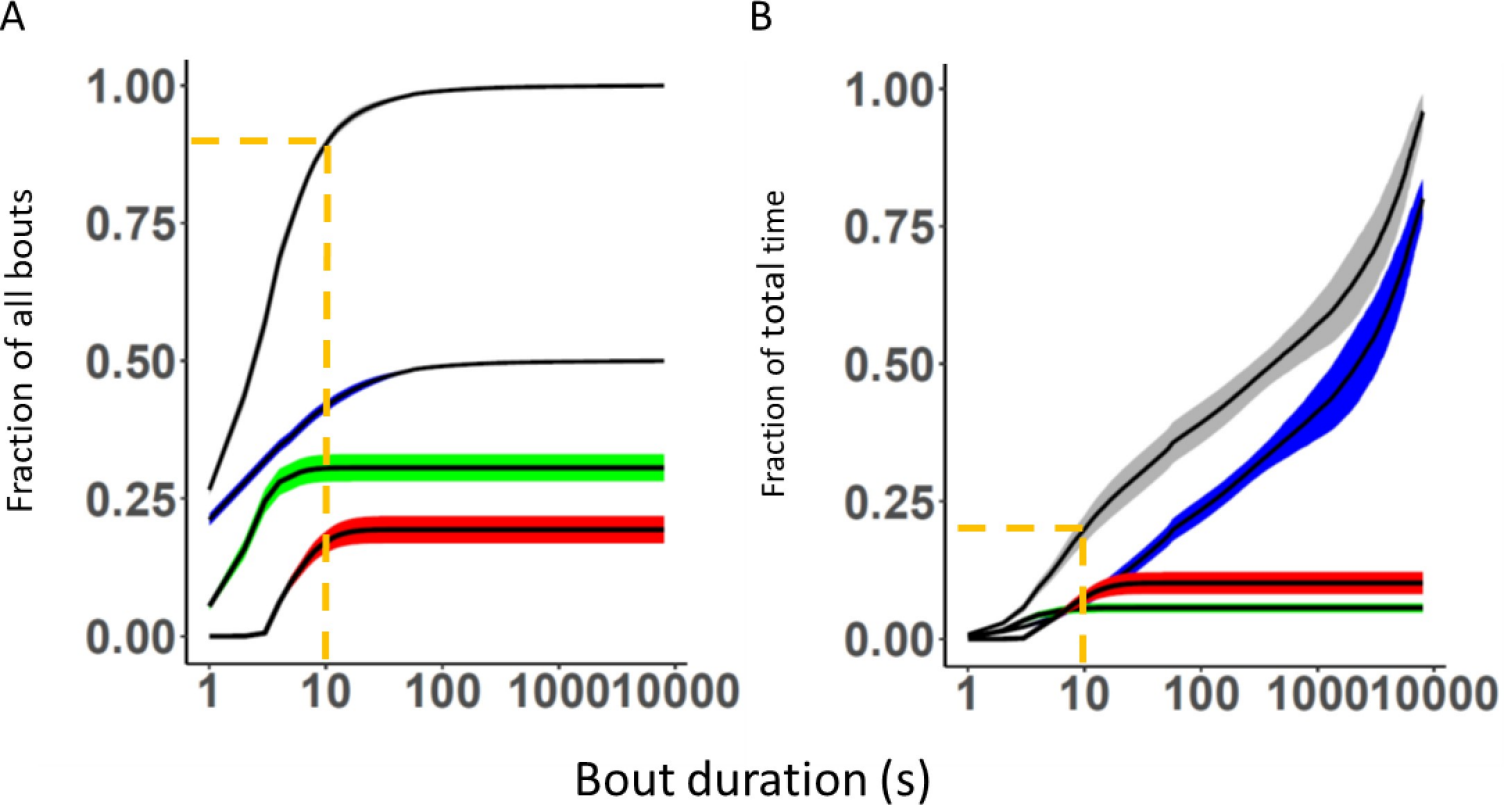
A Cumulative plot of fraction of the total number of bouts (ordinate) vs bout duration (s, abscissa, logarithmic scale) for all bout types (black line with SD as grey shaded area), rest bouts (black line with blue shaded area indicating SD), MOTS (black line over green area indicating SD), and locomotion bouts (black line on red shaded area indicating SD). B Cumulative percentage of total time (ordinate) plotted vs. bout duration (s, abscissa, logarithmic scale). Coloured area and code for bout type are the same as in A. Dotted line in A and B indicates number of bouts (˜90% in A) having a duration <10 s and their combined fraction of total time (<25% in B). A and B, data from single housed female mice.

A day in life of these ten mice is composed of a string of 6000–9000 bouts (˜0.06–0.1 Hz) of which 50% are rest bouts with median duration of 2.1s (Table 3 and Fig 3A and 3B). Two-point-four percent are long rest bouts with a median duration of 78s (mean duration: 343s; Table 3). Thirty percent of the bouts are short MOTS bouts (median duration 1.9 s; Table 3) while ˜20% are locomotor bouts with a median duration of 4.8s (Table 3 and Fig 3). Of the daily number of PA bouts, ˜75% occur during lights off. Thus, of all bouts in file >90% are PA and short rest bouts with a duration of ≤10s making up <25% of the file time (Fig 3A and 3B).

**Table 3:** 

Bouts of rest and movements					
	Median	Mean	SD	Range	
				Min	Max
Duration (s) [All rest bouts]	2.1	23.3	239.9	0.3	17165.1
Frequency of rest bouts [% of all bouts]	50	50	N/A	N/A	N/A
Duration of long rest bouts (≥40 s)	78	343	973	40	17165
Frequency of long rest bouts [% of all bouts]	2.4	2.4	0.4	1.7	3.0
Duration (s) [All activity bouts]	3.2	3.8	3.2	0.3	112.2
Frequency of locomotor bouts [% of all bouts]	50	50	N/A	N/A	N/A
Duration (s) of bouts of activity on-the-spot	1.9	2.2	1.5	0.3	66.1
Frequency of movement on-the-spot bouts	30.2	30.6	2.49	26.7	34.8
Duration of locomotor bouts	4.8	6.2	3.5	1.3	112.2
Frequency of locomotor bouts	19.8	19.4	2.49	15.2	23.3

**Fig 4:**
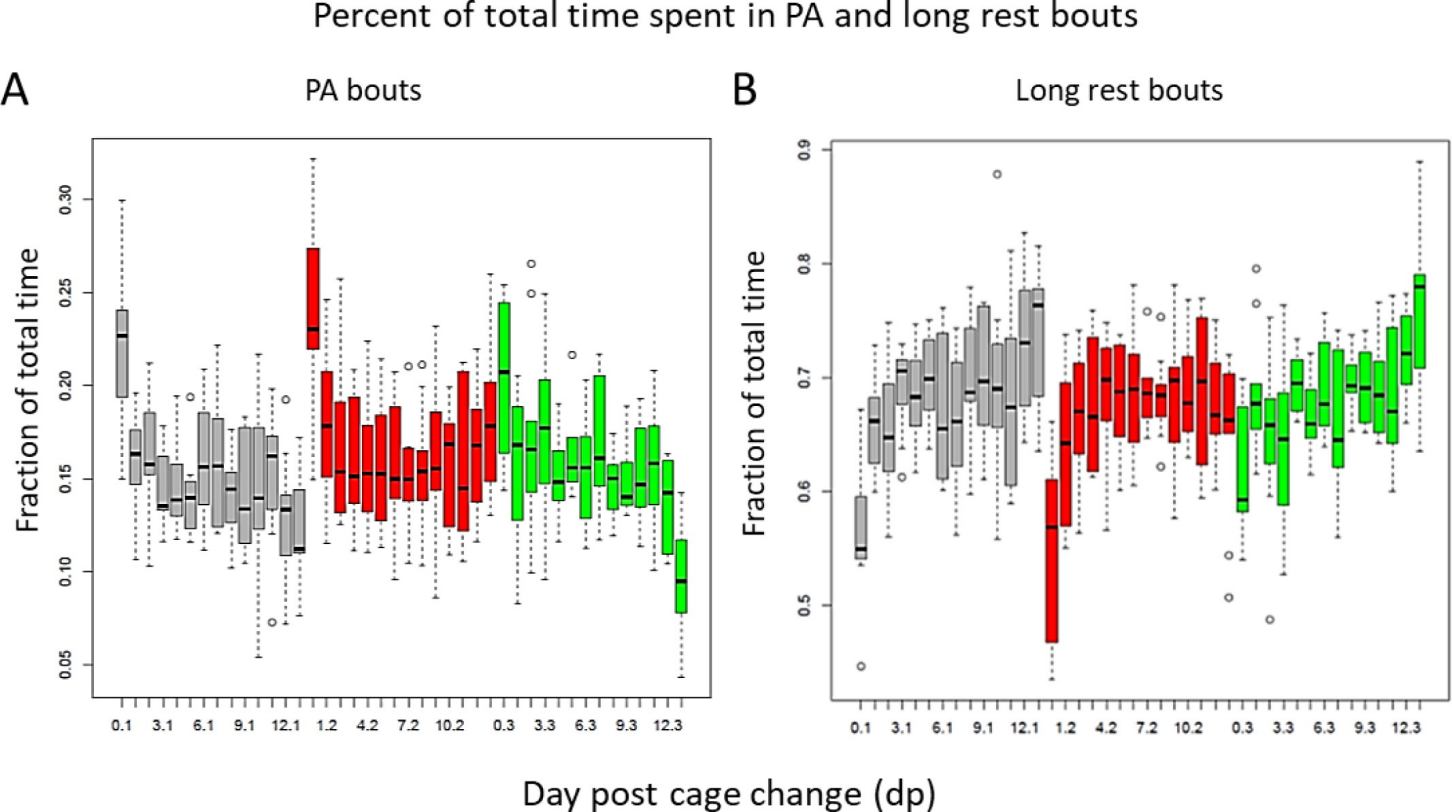
A and B show impact by day post cage change (dp 0–13; abscissa) and cage change cycle (CC 1–3; colour coded grey, red, and green) of fraction of total time spent in bouts of PA (A) and (B) long rest (ordinates). The relative effect size of dp and CC is shown in S5 Fig in S1 File. In A (model: PA ˜dp * CC), the major impact is by dp (p = 6.2E-11) with no contribution by CC (p = 0.16). B shows that CC has no significant impact on time in long rest (p = 0.54) while dp has a strong effect (p = 4.3E-11) (model: long rest˜dp*CC). A and B, data from single housed female mice.

**Fig 5:**
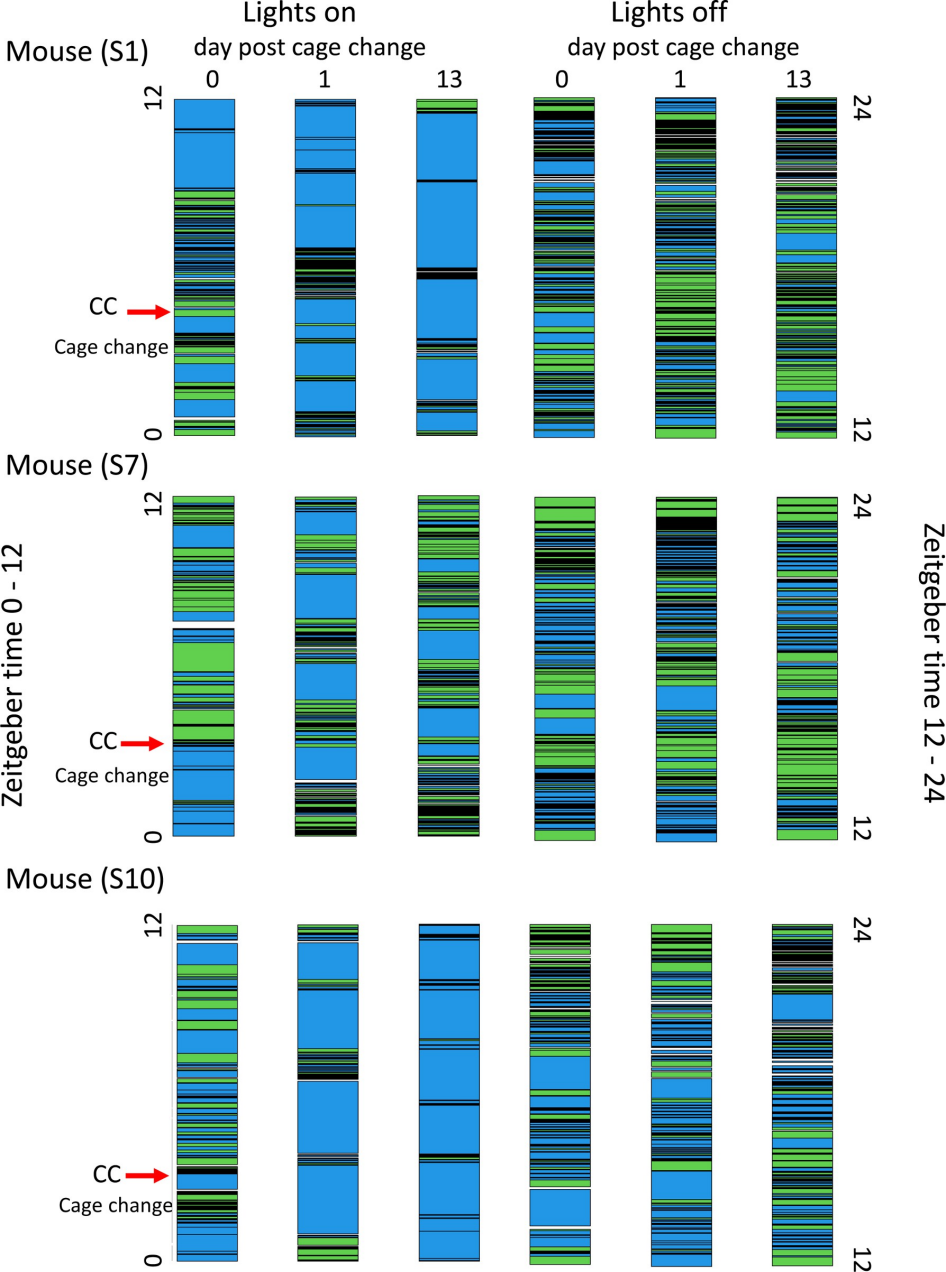
Pattern of long-rest episodes during lights on (ZT 0–12) and lights off (ZT 12–24) (DL 12:12) for the mice S1, S7 and S10 housed in isolation. Long rest bouts indicated by blue colour. Bouts disrupting long-rest periods in black/green. Ordinate is 12h lights on to the left and 12h lights off to the right. Columns are day post cage change (dp) where 0 is the day of the cage change (CC; red arrow indicates the time for CC) for lights on (to the left) and lights off (to the right). Dp1 the day after CC and dp13 the day before the next CC. See S6 Fig in S1 File for corresponding data of the other 7 single housed female mice.

**Fig 6:**
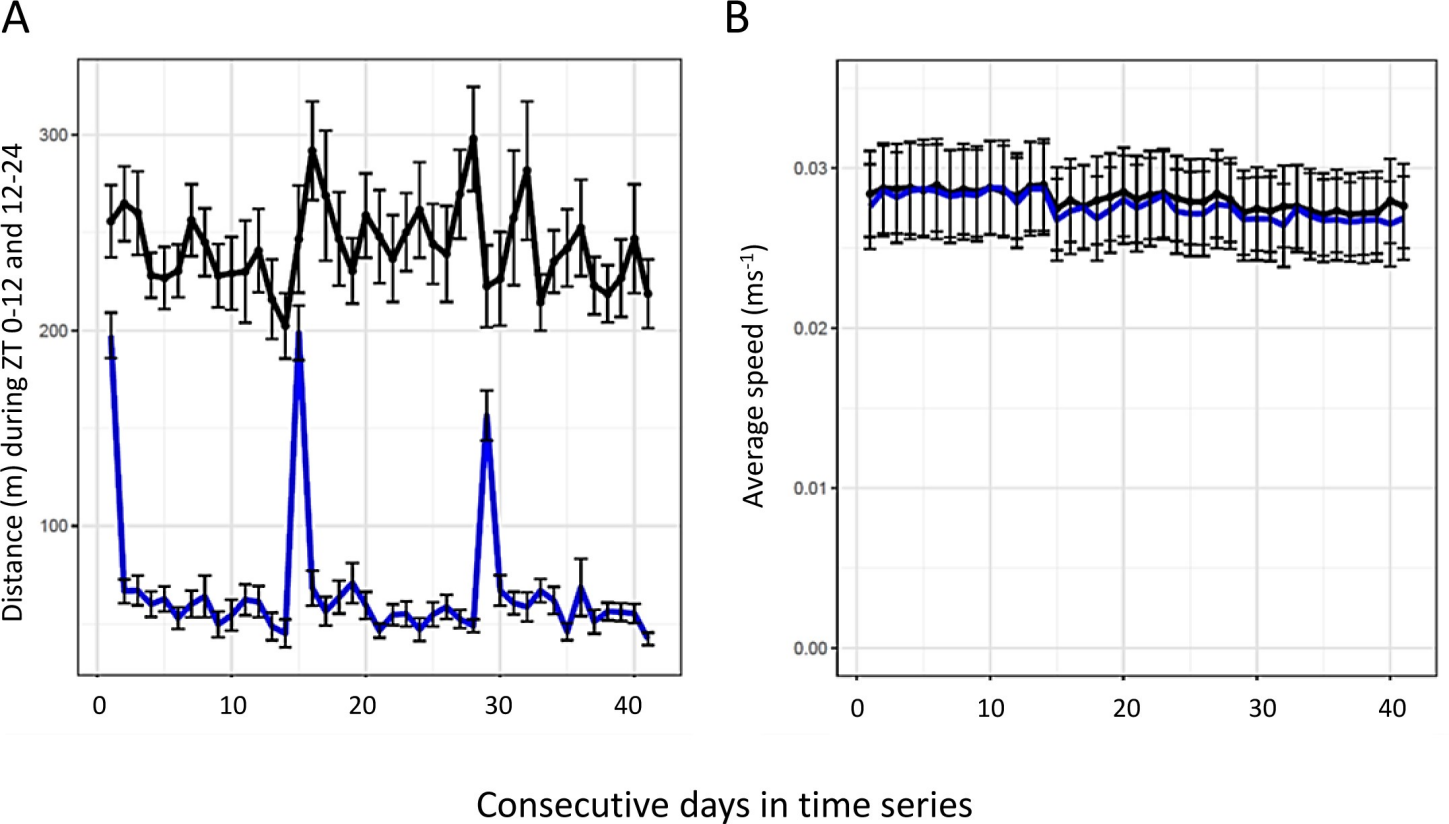
(A) shows cumulative distance per 12 hrs during lights off (black line) and lights on (blue line), respectively. Values are average across the 10 animals during ZT 0–12 and ZT 12–24 each day with standard error indicated by bars. (B) shows corresponding data for average speed (±SEM) during lights on (blue) and lights off (black). A and B, data from single housed female mice.

We confirm previous observations [12, 27] that the time in PA bouts decreases significantly 30 to -40% (p = 6.2E-11; Fig 4A) across the cage change cycle, while cycle-to-cycle variation was small (p = 0.16; see Fig 5A in S1 File). Conversely, the time in rest bouts drops initially followed by an increase by ˜30% (p = 4.3E-11; Fig 4B and Fig 5B in S1 File). The cage change also upsets the pattern of long rest bouts during daytime (lights on) (Fig 5; see also Fig 6 in S1 File). Days before a cage-change, long rest usually occurs as 4–6 bouts during day light [intercalated by bouts of PA and short rest] a pattern that is substantially fragmented by this intervention (*idem*).

### Distance and speed of bouts of motion

Bouts of MOTS are local and usually cover a Euclidian distance of less than 2.5 cm and motion is at low speed (Table 4). Locomotion usually covers 0.1 to 0.2 m, i.e., the distance from one side to the other of a cage and less than 1% of the locomotor bouts are longer than 1m (Table 4).

**Table 4:** 

Distance and speed made during bouts of movement					
	Median	Mean	SD	Min	Max
Locomotor bout velocity (m/s)	0.026	0.028	0.009	0.007	0.071
Locomotor bout distance (m)	0.139	0.174	0.118	0.065	3.726
MOTS bout velocity (m/s)	0.008	0.009	0.005	0.004	0.044
MOTS bout distance (m)	0.017	0.025	0.027	0.001	0.815

**Fig 7:**
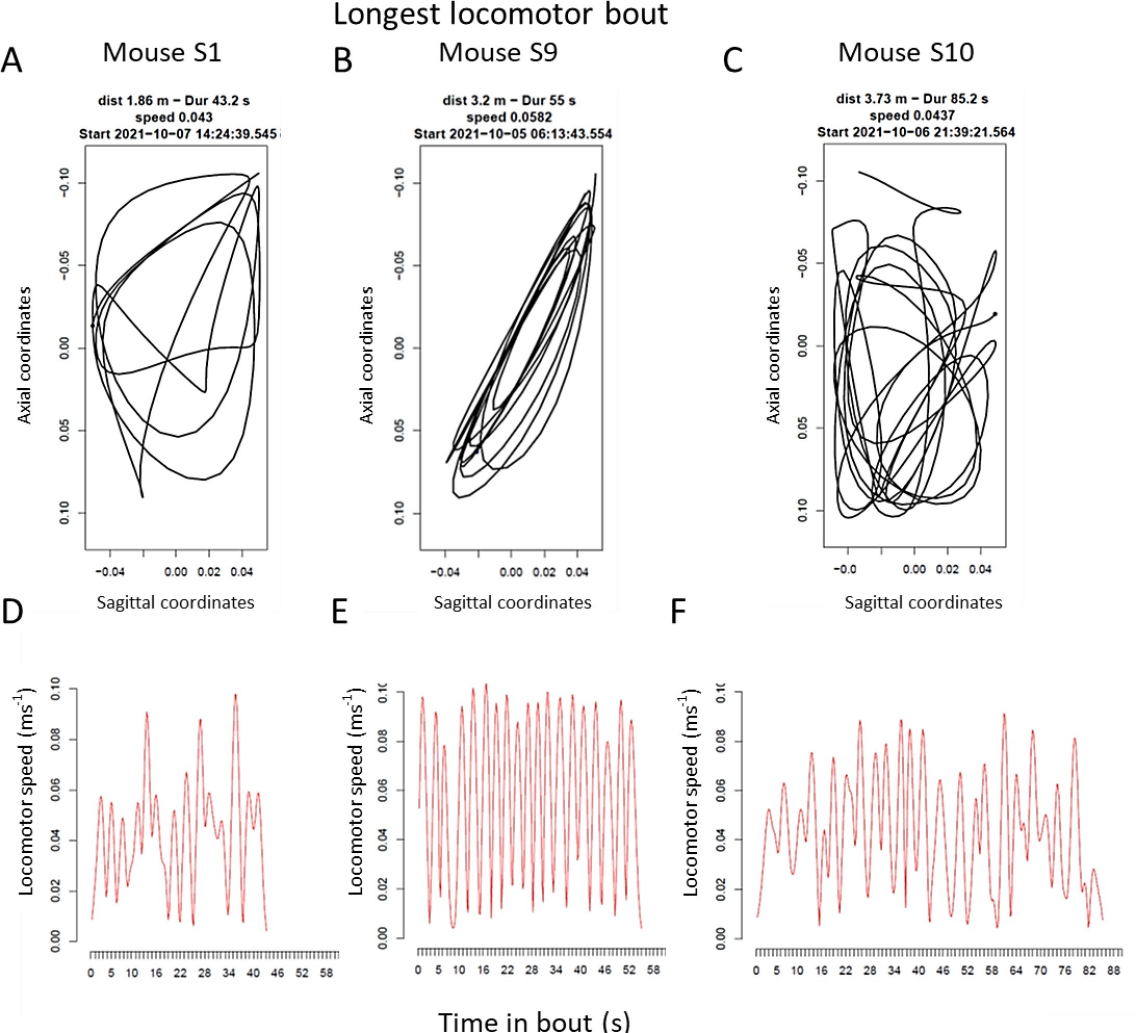
A-C show longest recorded locomotor bout for mouse S1, S9 and S10, respectively, during the observation period. In D-F, the corresponding speedograms are depicted. Track plots and speedograms by the trajar package in R. For tracks and speedogram of the other 7 single housed female mice see S8 Fig in S1 File.

Distance made per day is about 250m during the dark hours and about 80m during daytime, except for the day of the cage change (Fig 6A). The animal-to-animal variation in distance is larger during the dark vs. the light period of the day (*idem*; and Fig 7 in S1 File). However, the average speed during locomotion is very similar (˜2.8 cm s^-1^) in darkness and day light (Fig 6B and Table 4).

**Fig 8:**
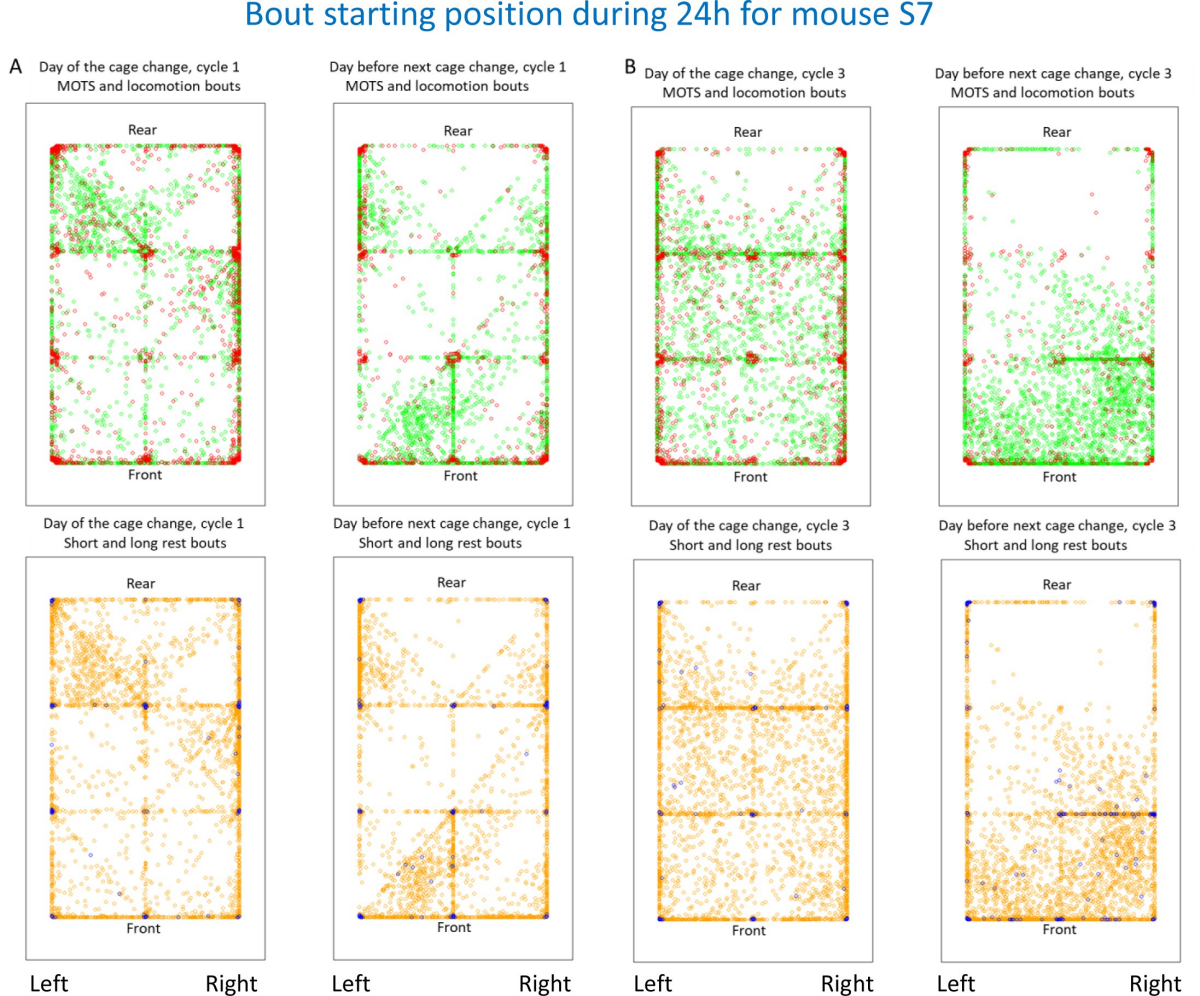
Densitograms showing the distribution of bout starting coordinates during 24h on dp0 and dp13 (columns) in cage change cycles 1 (A) and 3 (B) for the S7 mouse. Cage front and rear and left (L) and right (R) have been indicated. Top row of panels show MOTS (green) and locomotion (red) bouts, while lower row of panels show the corresponding data for short (orange) and long (blue) rest bouts. Please see S1 File for corresponding metrics of the other female mice kept in isolation.

Locomotor bouts covering longer distance (>1m; Fig 7A–7F and Fig 8 in S1 File) illustrates the variability of in-cage trajectories (Fig 7A–7C) and the speed dynamics of locomotor bouts (Fig 7D–7F). Speed ranges from a low of 0.01 m/s to 0.1m/s, and occasionally even higher speeds. Tracks also reveal occurrence of abnormal behaviours e.g., recursive locomotor activity (Fig 7B and 7E).

#### Spatial distribution of bouts’ starting point

**Fig 9:**
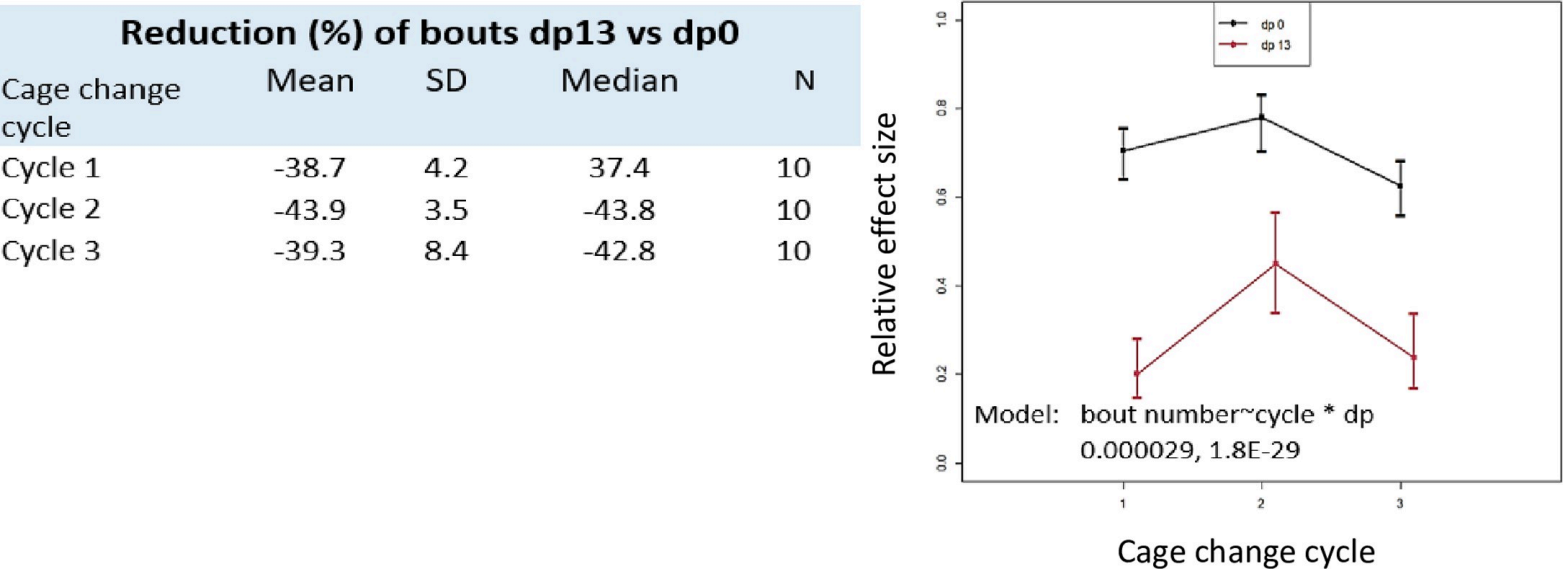
Reduction (%) of bouts dp13 vs dp0.

The starting coordinates for rest and PA bouts were analysed to explore how the cage floor is used and to compare the day of the cage change (dp0) with the final day of the cage-change cycle (dp13)(Fig 8 and Fig 9A–9C in S1 File). There is a considerable variability within and between mice, across a cage cycle and between cycles. Overall, there is a significant decrease by ˜40% (p = 1.8E-29; Fig 9) in total number of bouts/day across the cage-change cycle, while the variation in bout reduction between cycles was smaller (Fig 9).

In some cases (Fig 8 and Fig 9A–9C in S1 File) there is an even distribution across the cage floor of the starting points on both the day of the cage-change and at the end of the cage change cycle although number of bouts and their relative abundance changes (see also below). In other cases, bout initiation tended to be more clustered and this changed during as well as between cycles of the same mouse (e.g., case S7 in Fig 8).

**Fig 10:**
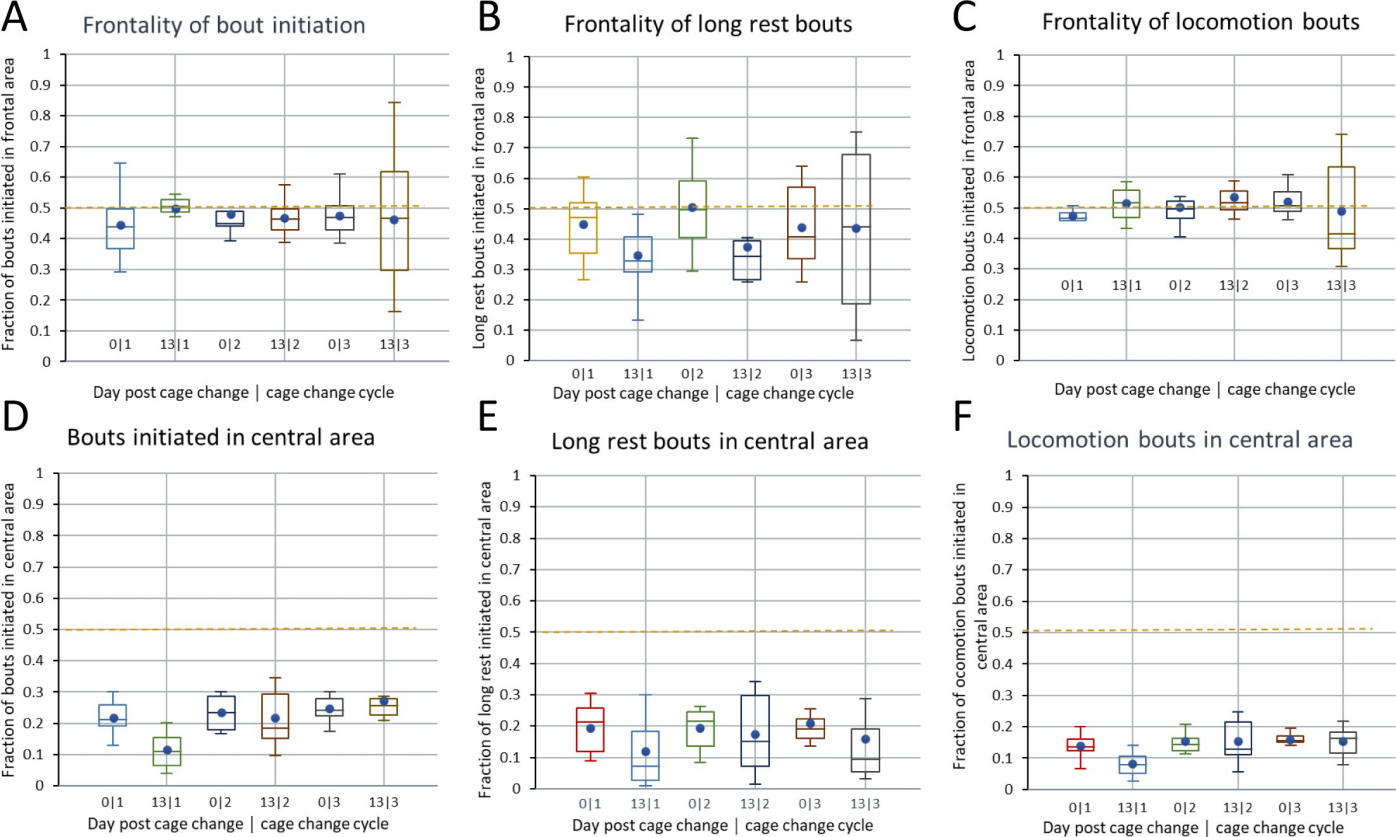
A Boxplots showing the preference for bout initiation in the frontal field of the cage floor on dp0 and dp13 through cage change cycles 1–3. In B and C, the relative frequency of long rest and locomotor bouts, respectively, starting in the frontal field of the cage floor have been indicated. D-F show the corresponding boxplots when the cage floor was divided into a central and peripheral field of equal size (see also Fig 1). A -C show data from single housed female mice.

There was no clear preference for initiating bouts in the frontal vs rear area of the cage floor (Fig 10A–10C; for definition of the cage floor sub fields see Fig 1), the distribution is close to even except for the few long rest bouts which tended to be more frequent in the rear (Fig 10B). In contrast, bout initiation was infrequent in the central area of the cage floor (˜25%; Fig 10D–10F), regardless of the bout being rest or a movement.

#### Rhythmicity of rest and locomotion of single-housed mice

**Fig 11:**
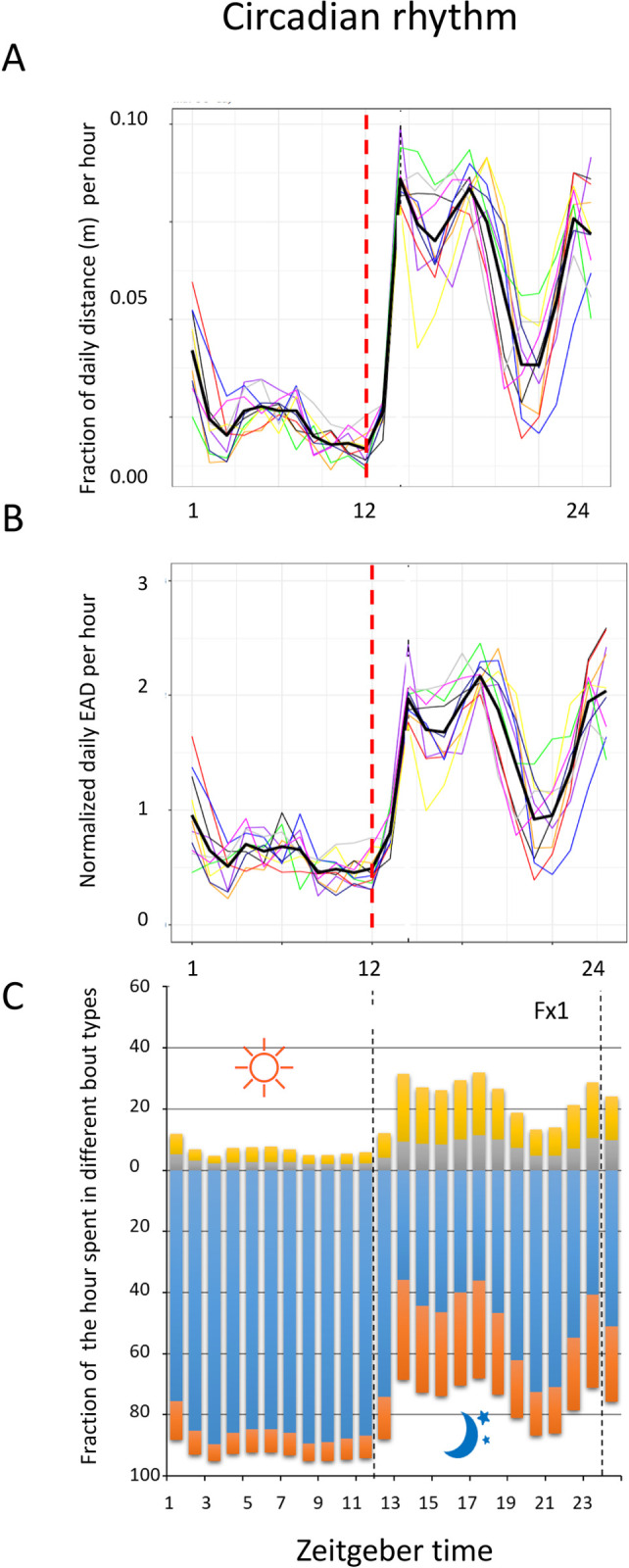
A shows fraction of daily distance (m) per hours during the LD cycle for each single housed female mouse (thin coloured lines) and the average across the ten cages (thick black line). B shows the EAD per hour when the animals are active during the LD cycle. Individual mice indicated by thin coloured lines, average across the group is the thick black line. C show the average fraction of each hour the mice spend in long rest (blue), short rest (orange), MOTS (grey) and in locomotion (yellow) across the LD cycle. Abscissa is Zeitgeber time with lights on 0–12 and lights off 12–24, the shift on to off is marked by interrupted vertical lines.

Mice follow a circadian rhythm of rest and PA, entrained to lights on and lights off in the laboratory environment (Fig 11A–11C; see also Fig 1 in S1 File). There is a nocturnal peak of PA while daytime holds the highest density of long periods of rest (Fig 11C). This pattern of rest and PA reproduces closely across cages (Fig 11A) and between metric used (tracking, Fig 11A; EAD, Fig 11B) across the LD cycle.

Decomposing the datafiles into bouts of long and short rest, MOTS and locomotion provide the basis for the observed alteration in travelled distance (or EAD) and EAD across the circadian cycle (compare Fig 11A and 11B with 11C).

### Activity and rest by electrode activation density (EAD) in single housed mice

**Fig 12:**
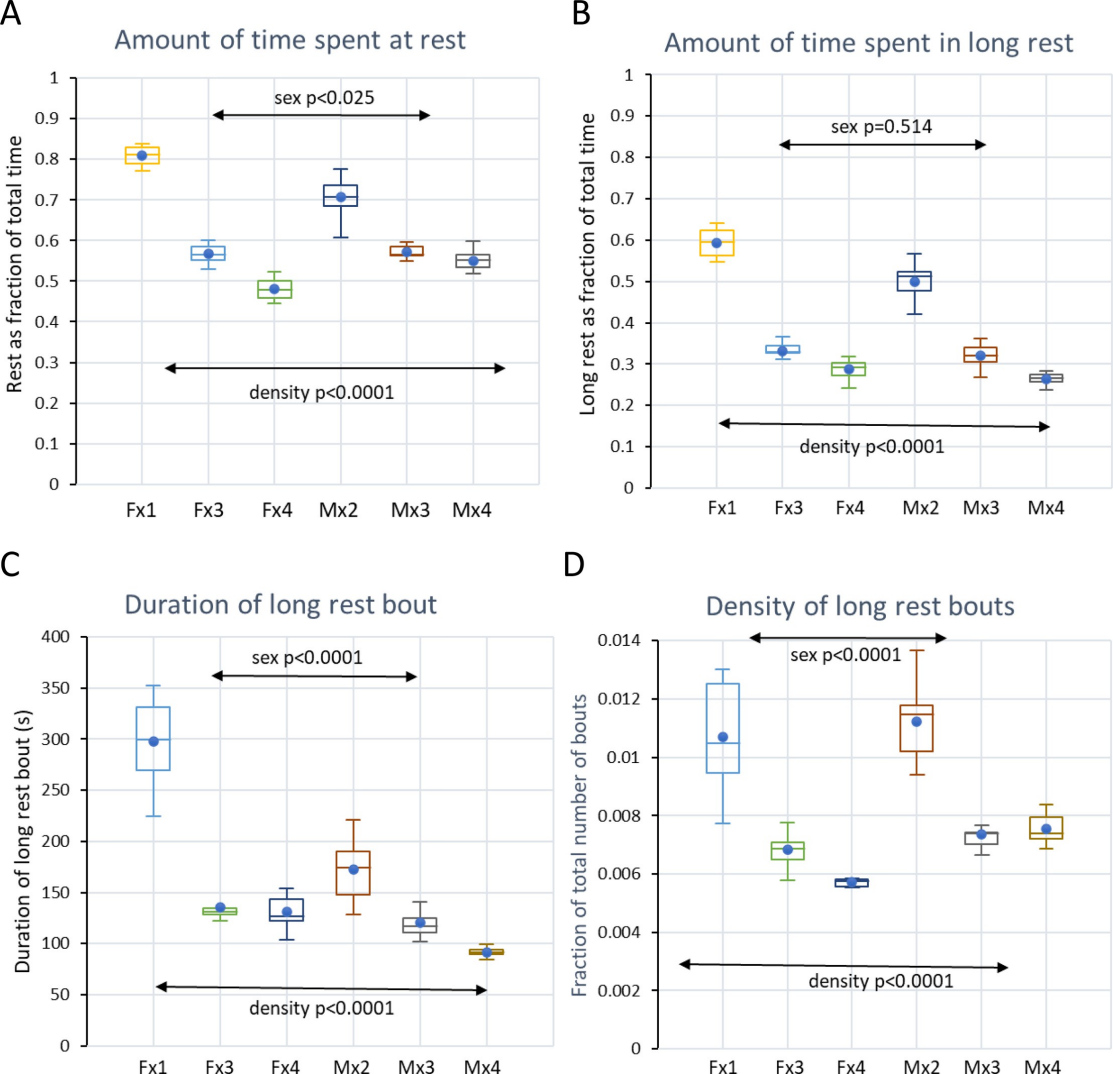
A-B Boxplots of fraction of total file spent at rest (no electrode activation) (A) and long rest (B) for the cohorts of female and male mice housed at different density (Fx1, Mx2, Fx3 etc). C-D show boxplots of duration and density of long rest bouts assessed by the EAD metric. As in A-B, female and male cohorts housed at different densities have been indicated. Statistics by ANOVA with model rest/long rest ˜ density*sex; significance of each factor has been indicated in the plots.

Consistent with the tracking data reported above, the EAD metric (see Material and methods) show that 73% of the time spent resting (81% of total file time) among single housed mice are bouts of long rest (≥40s; 59% of total time) having an average duration of ˜300s (Fig 12A–12C). The discrepancy in time spent in long rest bouts between the tracking metric and the EAD appears to be due to EAD occurring during bouts when the mouse centroid coordinates does not change (c.f. Table 2). Thus, EAD appears to be a more sensitive measure of the mouse being completely still than is the tracking metric.

### Comparison of housing density and sex by using time-series of EAD

While tracking can only be safely conducted on times-series recorded with a DVC of single-housed mice, EAD may provide some information on PA and rest when animals are group housed. As expected, increasing housing density decreases the amount of time spent in synchronized long rest (model: long rest ˜density * sex; whole model F = 239, p<0.0001; and p<0.0001 for density; sex was not a significant factor p = 0.514), mainly by reducing bout duration (Fig 12C, F = 130, p<0.0001; effect by density p<0.0001 and by sex, p<0.0001) but also the frequency (Fig 12D, F = 69, p<0.0001; effect by density p<0.0001 and sex p = 0.0001).

**Fig 13:**
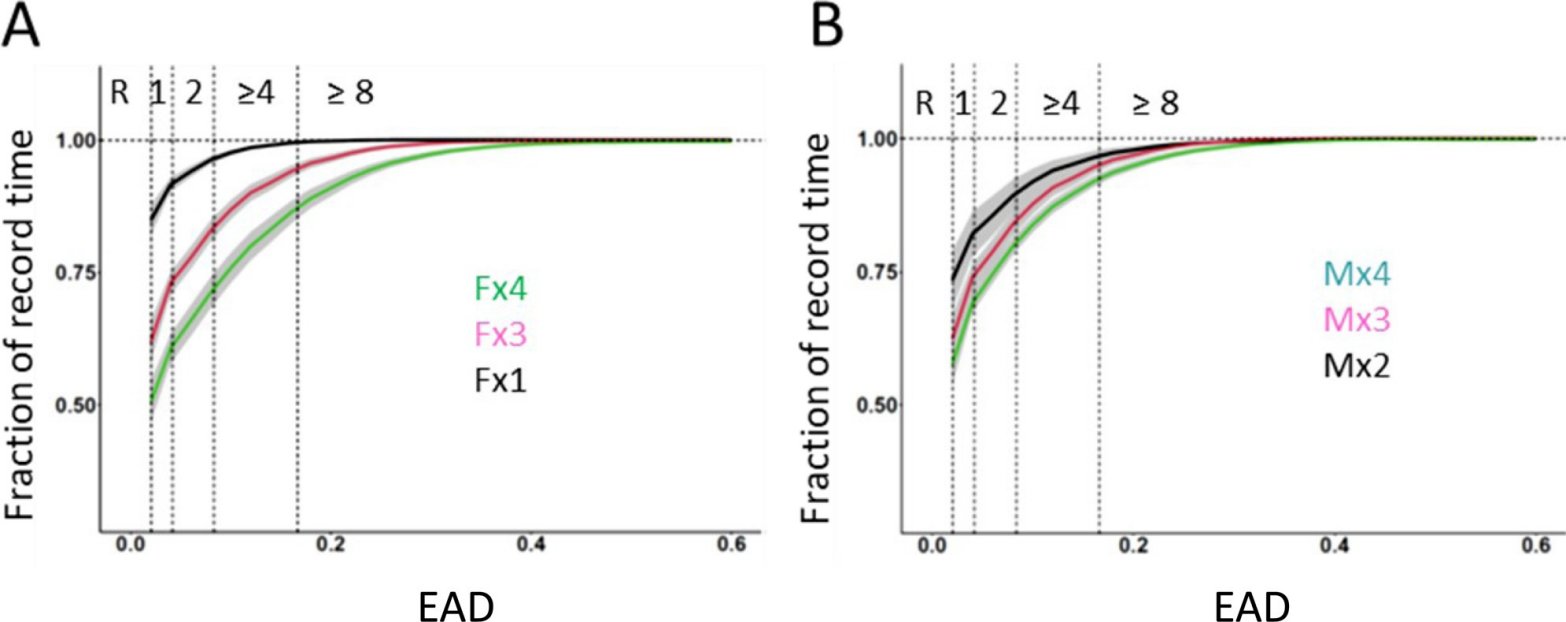
A-B Cumulative plots of time in PA assessed by EAD and the number of electrode activations observed (abscissa) as fraction of file time (ordinate) for each cohort of female (A) and male (B) cages (starting point of curve represent all bouts of rest (R). Housing density (x1, x2, x3, and x4) has been colour coded (key in panel A-B). Solid line represents cohort average value across cages and weeks of recording. The shaded area with the same colour indicates the standard deviation. Interrupted vertical lines indicate cut point values for rest (R) and change in electrode activations s^-1^from one, two, four and eight or more electrodes activated.

In Fig 13 the impact of housing density and sex on time in PA (EAD) is shown and the effect size (Hedge’s q) has been tabulated in Table 5. The stepwise increase is large (effect size˜2) and without any apparent difference between sexes at density x3 (Table 5). The effect size on time in PA (EAD) by increasing density from x3 female or x3 male mice up to x4 male mice was 1.4, which is only half of the effect size (˜3) recorded for the step up from x3 male or female mice up to x4 female mice (Table 5).

**Table 5:** 

Effect size of sex and density on PA as % of file time							
Cohort name		fx1 (n = 1)	mx2 (n = 2)	mx3 (n = 3)	fx3 (n = 3)	mx4 (n = 4)	fx4 (n = 4)
fx1 (n = 1)	Hedges g		2.01				
mx2 (n = 2)	Hedges g			2.10	2.22		
mx3 (n = 3)	Hedges g				0.09	1.40	2.90
fx3 (n = 3)	Hedges g					1.46	3.05
fx4 (n = 4)	Hedges g					1.89	

### Rest and activity (EAD) across the LD and the cage-change cycle

**Fig 14:**
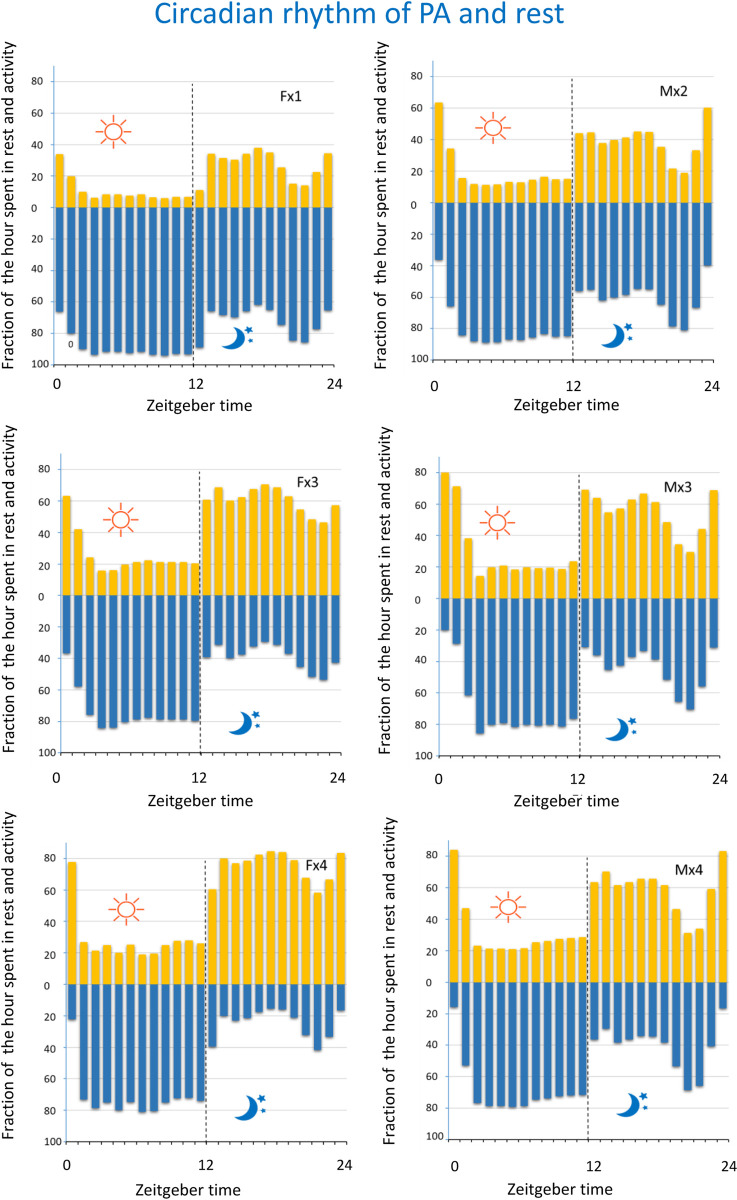
Panels show the average fraction of each hour spent in PA (yellow) and at rest (blue) across the LD cycle for male and female mice housed at different densities. Rest i.e., no electrode activation and PA when electrode activations occur. Abscissa is Zeitgeber time with lights on 0–12 and lights off 12–24, the shift on to off is marked by interrupted vertical lines.

**Fig 15:**
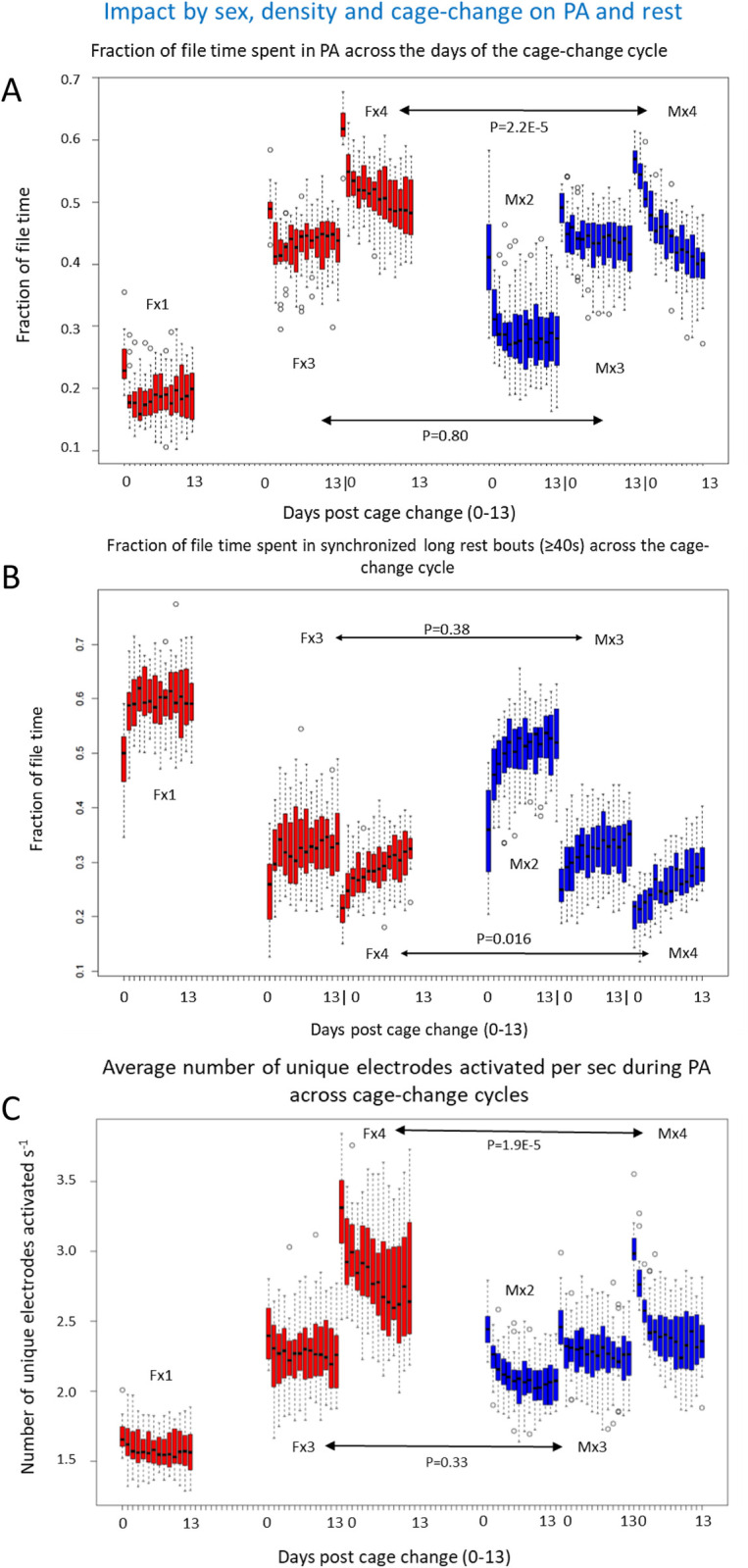
A show fraction of file time spent in PA (≥ 1 electrode activated s^-1^) across cages per day (dp) of the cages change cycles 1–3 for male and female mice housed at different densities (x1: n = 1, x2 n = 2, x3 n = 3 and x4 n = 4). Comparison of female (red) and male (blue) mice at density n = 3 and n = 4, respectively, revealed a significant difference at density n = 4 but not when density = 3 (model: Time in PA ˜ sex * dp * CC; density = 4 F = 36.6; n = 3 F = 0.07). See also Fig 10 in S1 File for plot of relative effect size of dp and CC for males and females at densities n = 3 and n = 4, respectively. B show fraction of file time spent in long rest bouts (<1 electrodes activated s^-1^ and≥40 s duration of bout) across cages and cages-change cycles (CC 1–3) per day (dp0-dp13) for male (blue) and female (red) mice housed at different densities (x1 to x4). Comparison of female and male mice at density n = 3 and n = 4, respectively, revealed a significant difference in synchronized long rest bout time at density n = 4 but not when n = 3 (model: Time in long rest ˜ sex * dp * CC; n = 4 F = 7.6; n = 3 F = 0.79). For plot of relative effect size of dp and CC at these densities see Fig 10B in S1 File. C show average number of unique electrodes activated s^-1^ across cages per day of the cages change cycles 1–3 for male (blue) and female (red) mice housed at different densities (x1 to x4). Comparison of female and male mice (model: No unique electrodes ˜ sex * dp * CC) revealed a significant difference at density = 4 (F = 41, p = 1.9E-5) but not at x3 (F = 1; p = 0.33). For plot of relative effect size of dp and CC at these densities see Fig 10C in S1 File.

As previously reported the amount of PA and rest per hour varies systematically across the LD cycle (Fig 14) and the proportion of synchronized-rest decreases as housing density increases. Above we showed that bouts of rest and PA of single housed mice were impacted by the cage change (Figs 4 and 6) affecting both distance moved and bout composition. When we use EAD as metric (Fig 13 and Table 5) we obtained corresponding results for the single housed mice and a stepwise increase of effect size as we increase housing density up to four. In line with the results using aggregated EAD data (Fig 13A and 13B and Table 5), there was a significant difference between sexes at density x4 but not at x3 with respect to both time spent in PA and time of synchronized rest (Figs 13B and 15A). In all the different housing densities studied here, there was a marked impact by the cage change on time spent in PA and synchronized-rest, respectively, in both sexes (*idem*; for effect size See Fig 10 in S1 File).

EAD increases (Fig 15A and see also Fig 3 in S1 File) with housing density and in parallel a larger number of unique electrodes are activated during a PA bout (Fig 15C), suggesting that number of unique electrodes involved in a PA bout covariate with EAD.

**Fig 16:**
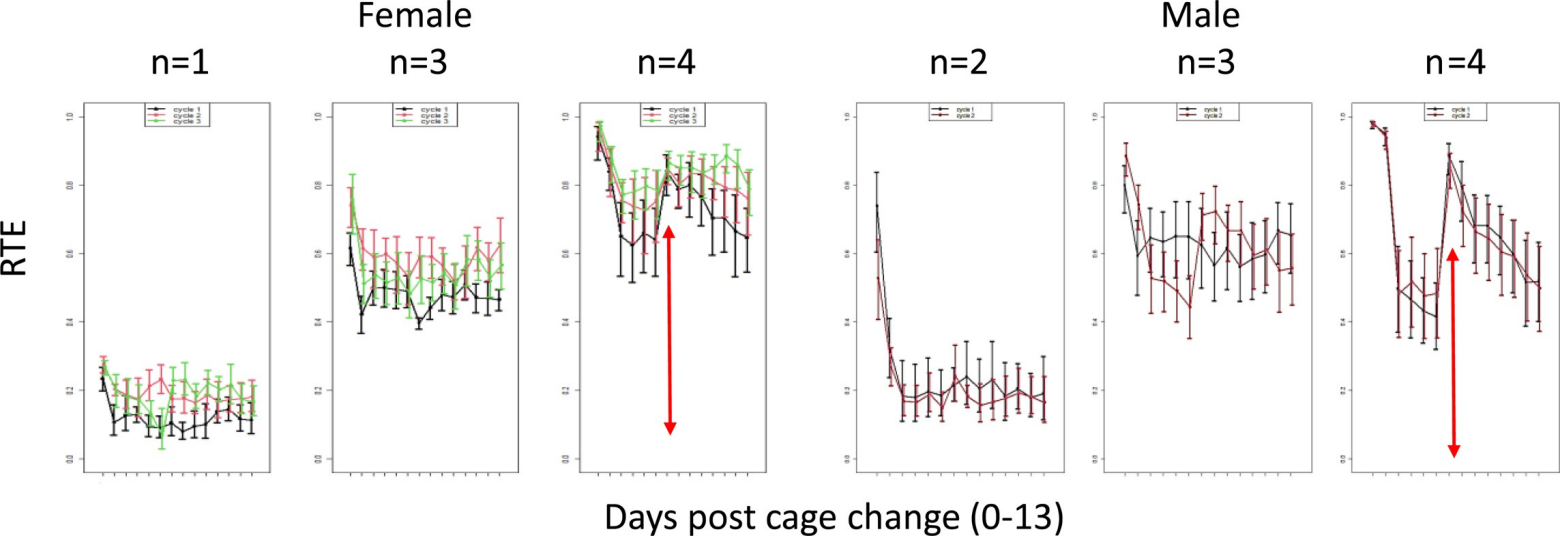
The panels left-to-right show relative effect size (RTE, ordinate) on average daily EAD by housing density (n), days post cage change (abscissa; dp0-13) and cage-change cycle (CC 1–3; colour coded black, red, and green, in each panel) within sexes (group of panels). Whole model: EAD ˜ N * dp * CC; for females F = 163.7, p = 7.6E-15; for males F = 28, p = 3.3E-7. In both sexes, density (n) had the strongest impact on EAD, followed by dp, while impact by cage change cycle was only significant in females. NB: In males only two cycles could be compared across densities. Note that at N = x4 EAD show a biphasic trajectory across all cycles in both sexes (red arrows, for further information see text).

With a housing density of four female or male mice, the relative effect size of days post cage change on PA and rest across the cage change cycle displayed a biphasic trajectory with a second distinct infliction on the day when neighbouring cages were subjected to a cage change. Such inflictions were occasionally also evident in cages with three animals but not a consistent feature through cycles (*idem*) and, furthermore, not seen with lower housing densities (Fig 16).

EAD occurrence in the frontal vs the rear part of the cage floor was rather similar with 5%-10% more EADs in the rear of the single-housed mice. In two out of three cycles, the percentage of EADs in the frontal area increased by 5–10% towards the end of the cage change cycle (Table 6).

**Table 6:** 

	Frontality of EAD during and across cage change cycle(s)							
	dp0			dp13			Δ % dp0-dp13	
	Mean	Median	SD	Mean	Median	SD	Mean	n
Cycle 1	41.7	38.4	11.3	48.0	48.6	8.1	6.3	10
Cycle2	44.0	41.6	9.8	52.5	50.6	10.4	8.5	10
Cycle3	47.9	49.9	8.2	45.6	45.7	19.4	-2.3	10

## Discussion

### General comments

In this study we use decomposition analysis of longitudinal data recorded with a DVC HCM system covering six weeks to assess pattern of rest and PA of single and group housed C57BL/6J mice. The decomposition of the files recorded with the tracking function in cages with single housed female mice was based exclusively on the occurrence or absence of a change between samples of the mouse centroid position (≥1 mm). Further subdivision of bouts were based on bout duration (short and long rest, split at 40s) and with PA bouts into MOTS (local movement) and locomotion (the bouts’ radial distance was larger, or equal to, one average stride length for this strain and sex) [17, 19]. Thus, the decomposition into bouts did not compromise the resolution of the system. In single housed mice, we extracted both track and EAD metrics from the raw data to assess degree of covariation. As confirmed here (see [16]), EADs and distance by tracking per hour correlate closely but there is not a perfect match (˜3% of file time differed). With tracking it is straight forward to differentiate between MOTS and locomotion, however, associating EAD with number of different electrodes activated and the temporal succession of electrode activations during a bout, may prove useful as guidance to separate locomotor and MOTS bouts with the EAD metric. As with tracking, however, this can only be applied when animals are single-housed [15, 16]. In the discordant segments of the aligned [EAD and tracking] files, electrode activations were recorded during periods when the tracking did not recognize any movement (<1mm, ˜resolution of the system [15]), i.e. during bouts of rest. Although this deviation between records is only a few percent of the total file time, the metrics on time spent in long rest differed (EAD: 59% and tracking: 67% of recorded time) while the difference of total time in rest was only ˜3%. A plausible explanation is that EAD is a more sensitive metric of PA, possibly responding to changes in body posture and movements of the tail. Such electrode activations may not be sufficient to alter the x, y position of the mouse centroid. This led us to interpret long rest bouts as sleep only if both metrics indicated that the animal did not move (see also [21] on criteria stringency of being still). Thus, the time budget for rest of single housed (C57BL/6) mice was ˜81% considering both metrics extracted, and sleep (NREM+REM) 59% (based on EAD). With the definition for PA bout types used here, about ˜6% is used for MOTS and ˜10% for locomotion.

With DVC, the tracking metric is more informative than the EAD but we recommend to extract both and to use them in parallel (as we did here to define rest as sleep). EAD can also be used when animals are co-housed to relay information about bouts of synchronized-rest and group PA (density and spatial distribution of activation) of the animals (see also below) and have been used to monitor circadian rhythm, impact by husbandry routines, and progression of biological processes and diseases [12, 27, 29, 33, 42, 43]. To monitor level of in-cage PA may be helpful in surveying the recovery of e.g. surgery or response to interventions to impede ageing and other biological processes (*idem* and [44]).

### Bouts of rest and PA

Single housed mice generate ˜7100 bouts of rest and PA per day, this is in agreement with data for C57BL/6 obtained by a HCM system using force plates [45]. Fifty percent are bouts of PA corresponding to only ˜16% of the file time (see also [46, 47], while only 2.4% of the bouts are long rest bouts considered sleep episodes making up 59% (by EAD) and 67% (tracking), respectively, of the file time. Sleep is divided into non-REM sleep (NREM; in mice >90%) and REM sleep (REM; <10%). The gold standard to decide on state of vigilance is to record gross cortical brain activity by surface electrodes (EEG), eye-movements, and muscle tone (EMG). Pack et al [48] found by comparing EEG and EMG with video recordings a >90% agreement between long periods of rest (CCD; ≥30s) and EEG-EMG pattern of NREM and REM sleep. These observations were later confirmed and extended by correlative assessment of home-cage periods of long rest using piezo-electric sensor [49], IR-sensor [20, 50], CCD [21, 22], and electric-field sensor [51] based HCM techniques combined with EEG-EMG recordings. The results of these studies show a correlation >0.9 between NREM and REM sleep, on the hand, and the animals being still ≥40s, on the other. This behavioural criterion for sleep has subsequently been used in a number of studies [52–56]. We conclude based on our results on single housed female mice and those obtained with other HCM techniques (*idem*) that bouts of inferred sleep (no distinction made here between NREM and REM) in single housed mice of this strain commonly have a duration of 300-400s but can exceed 1000s and occur with an average density of ˜120 bouts per day preferentially during lights on. Moreover, our bout actigrams revealed that sleep bouts were clustered at 4–6 time periods during lights on, and 1–2 episodes during lights off. Interventions like a cage change induces considerable fragmentation of bouts, affecting those considered to be sleep (see also [28, 57–59]).

With co-housing the time spent in synchronized long rest (no EAD; all animals at rest) decrease, in pair housing to 45–50% of file time, with trios down to ˜35% and no difference between sexes. Still at a housing density of four mice per cage ≥25% of the file time is spent in synchronized long rest, inferred to be sleep. At this density, males had significantly shorter synchronized long rest than females.

As highlighted by Golani and co-workers [17], bouts of physical activity in mice can be divided into locomotion and local movements (MOTS; see also [45, 46]). Local movements comprise a range of behavioural entities not possible to decode with the DVC system. These entities include feeding, drinking, rearing, and grooming (*idem* and [47]). MOTS make up ˜60% of the PA bouts and 38% of the file time devoted to PA. They are more prevalent during lights off (˜65% of all daily MOTS) and in agreement with data recorded with other types of HCM systems [45, 46] they usually have a short duration (≤2s), and cover typically a short distance (<2cm) at low speed (<1 cm s^-1^). MOTS bouts increase in response to a cage change and in the responses to lights on/off during the LD cycle, and cover a cumulative distance of 40-70m per day, i.e. ˜15–20% of the daily moved distance (see also [45]).

We recorded 1000–2000 bouts of locomotion per day in single housed mice. As expected, few occurs during lights on because mice are nocturnal. The typical locomotor bout covers a distance of 0.1–0.2 m with a speed of ˜3 cm s^-1^, which agrees with previously published data recorded with DVC and other types of HCM systems [54, 57, 60, 61] but lower than those reported by [45, 46]. This discrepancy in average speed may relate to differences in the definition of a locomotor bout (speed or distance). As shown here, speed varies (range˜0.01–0.1 m s^-1^) during a locomotor bout, and the trajectory and bout speedogram will unmask abnormal recursive motor behaviours (fits of stereotypy). Distance travelled per hour is in the range from <1m h^-1^ (day time during periods of rest) up to 40m h^-1^ (in response to lights on, and cage change), decays across days of the cage change cycle and show considerable differences (one fold) between [single housed] mice. The daily average distance covered by the mice was ˜330m in this study, which is within the range (˜150–750 m 24h^-1^) of previously published data [16, 45, 46, 57, 60, 61]. The difference noted in average speed between our results, including that we did not find a difference in speed during movements day time vs night time, and those previously published using DVC is due to that we used decomposition into bouts, while Iannello’s [16] data is average across all movements and rest per unit time, and Shenk et al. [61] used a different definition of locomotion.

### Rest and PA across the LD cycle, and the use of the cage floor

In mammals, the pattern of PA and rest follows different rhytmicities (for references see [29]). Universal is the circadian rhythm which entrains to Earth’s Day and Night (*idem*). HCM systems are ideal for the purpose of analysing behavioural rhytmicities over extended periods of time in small rodents [29, 33] and may prove to be a good substitute to the current gold standard of using running wheels in studies of the circadian rhythm [62]. Mice are nocturnal and will rest during day time (lights on) while they are active and feed during night time (lights off). Our data show that across the LD cycle, the driving force is the clustering of long rest bouts to daytime, while all other bout types increase in prevalence during night time. We also confirm that the responses to lights on/off appears insensitive to if lightning change suddenly or through a dawn and dusk transition period [27].

In a previous report we showed that the timing of the response to lights off in female C57BL/6J can differ between facilities despite using the same sudden change regime [12] and in a recent multicentre study [27] we could not see a difference between sites in PA or circadian pattern between those using dawn and dusk and those using a sudden change in lightning.

Although less informative than tracking EAD revealed the same pattern of rest and PA in single housed mice and, further, that the amount of time spent in PA bouts increase stepwise up to a density of four mice [for the C57BL/6J strain of mice]. At housing density of three mice per cage we could not detect any sex difference in the pattern of PA and synchronized rest while with 4 mice to a cage, the level of PA and rest differ between sexes [12, 27].

In a recent publication we showed that male mice housed 4 to a cage tend to constrain the use of the cage floor area across the cage change cycle and that this may be related to the location of the in-cage latrine(s) [27]. Females at the same housing density did not show the same degree of spatial clustering of PA and, importantly, this was not evident when males were housed in pairs (*idem*). Single housed mice, tend to locate long rest into the rear half of the cage (mainly during lights on), while all other bout types (97.6%) were spread across the front and rear of the cage floor but with a significant portion of them (75%) originating (and terminating) in the peripheral part (50% of floor area) of the cage floor. Thus, housing C57BL/6J mice at densities ≥4 per cage seems to alter in cage behaviour and induce sex differences, combined this may indicate a crowdedness stress that should be taken into account when designing experiments and comparing study results.

### Decomposition of longitudinal HCM records into bouts of rest and PA may have significant value in translational research

The introduction of small wearable devices (accelerometers, more recently smart watches and similar items) that can be carried on the wrist, thigh, or trunk without disrupting normal activity has made it possible to collect large amounts of longitudinal data on rest and activity from healthy and sick, growing and aging humans, to extract metrics useful as objective biomarkers of development and ageing, disease progression, outcome prediction as well as monitoring impact by intervention [34, 63–76]. So far, only few accelerometer studies on humans encompasses variation in activity and rest bouts across the circadian cycle [34, 64, 68, 69, 73]. However, as smart watches and similar devices are likely to replace the older more unpractical accelerometers this will likely change. The number of papers by year presenting accelerometer data in humans has increased x25 from year 2001 to 2020 (source PubMed). Work is ongoing to develop recommendations to standardize accelerometer records as well as new tools by which the data can be analysed [31, 32, 64, 77–83]. Similar initiatives are currently ongoing in the realm of HCM of animal models in the life sciences with a recently started COST action in EU (TeaTime) and the North American 3Rs collaborative (Na3RsC).

The most common approach so-far to analyse accelerometer/smartwatch raw data is by decomposition analysis using cut-point values to stratify the data into bouts of sleep (SL) and sedentary behaviour (SB), low-medium and vigorous physical activity (PA) or different combinations of these categories (*idem*). The frequency and duration as well as accumulation pattern of different bout types and the composition of bouts are then compared over time and/or between groups. With GPS tracking becoming a more frequent feature of wearable devices also distance made and speedograms will be possible to retrieve as indices. Similarly, data generated by a variety of HCM systems have recently been used in efforts to identify behavioural indices of ageing [29, 84], impact of disease progression [42, 43, 61, 85, 86], genetic modification [45, 49, 52, 54, 87], and insults [60, 61, 88].

Since the data generated by wearable devices in humans and by non-intrusive scalable HCM systems in animals essentially overlap, it should be feasible to agree on sets of metrics that will serve as equivalent biomarkers for different conditions and biological process in both humans and small rodent models used to study human conditions.

### Limitations of this study

With few exceptions [9, 89, 90], scalable HCM systems such as the DVC used here can provide detailed metrics only when animals are kept in isolation (for references see Introduction and above). Mice are normally living in groups and as stated in the EU Directive63/2010 there are ethical reasons to avoid single housing of laboratory rodents, since evidence indicate a depreciation of animal welfare and that isolation may alter animals’ mental capacity and spontaneous behaviour [91–98]. However, some studies especially on male mice have questioned this and showed that the welfare or behaviour must not always be depreciated and, furthermore, depends on the context [96, 99–103].

To enable assessment of data extracted from the raw electrode output of the DVC, we used a cohort of single housed females as the main subjects of this study. Our results indicate that with co-housing, PA (EAD) increased in a stepwise fashion from single housing to pair housing and from pairs up to trios, for this strain of mice. Thus, our data suggests that welfare depreciation experienced due to single housing did not seriously affect the mice’s daily amount of PA to a significant degree. It remains roughly proportional to number of animals in the cage up to a density of four animals.

Both the spatial (12 electrodes spaced apart) and temporal resolution (4 Hz) of the system are low compared to video-based solutions (usually ≥ 15 FPS and HDMI) but has the advantage of low demands on IT infrastructure, data storage and processing capacity. Except for the rather few long bouts of rest, the different bout types had a frequency in the range of 0.05–1 Hz. Thus, the over-sampling at 4 Hz should be sufficient to delineate the bouts accurately. The HCM system is fully automatable, scalable, and non-intrusive. Similar, to RFID (Intellicage, TSE; e.g. [4]), IR-beam (Actimot, TSE; e.g. [8]) and Force plate (Metris, Labora; e.g. [6])-based systems, the output does not have high dimensionality. Thus, this type of system cannot capture behavioural trait details, only basic metrics as animal localisation, rest, and PA. An additional limitation is that recording of PA (and rest) with the DVC system is restricted to the floor of the cage and activities such as e.g. climbing is not recognized. Moreover, it cannot be excluded that data of PA was not accurately picked-up when the animal was on top of shuffled piles of bedding and enrichment materials.

Still, as shown here tracking and EADs can be quite informative when the animals are kept in isolation, while housed in groups the information provided (by EAD metric) apply only to the group not individual animals.

### Concluding remarks

We show that data on a variety of parameters such as sleep pattern, locomotor activity, bout fragmentation, spatial distribution of rest and PA, the circadian rhythm and changes to these metrics induced by interventions, can be extracted in near real-time from scalable HCM systems like the DVC using standard desk top computers. This information is certainly of value to assess in-cage animal welfare and health (e.g., sleep fragmentation, bouts of abnormal locomotor behaviour) as well as responses to husbandry and other holding conditions. It provides a menu of objective biomarkers to assess various experimental interventions as well as phenotypic changes by genetic manipulations. Longitudinal data can easily be generated and retrieved on a large scale serving both the care and welfare of the experimental animals, and the research conducted on them. The main bottle neck is that animals need to be kept in isolation. With group housing the output is less informative and applies only to the group of animals in the cage but may still be valuable for welfare surveillance and experimental monitoring. Finally, the output from these systems compares well with data generated by wearable devices on humans and may, thus, form a basis for translatable behavioural biomarkers.

## Supporting information

S1 File (PDF)
