# Supplementary material for: Bouts of rest and physical activity in C57BL/6J mice
Source: PLoS One. 2023 Jun 26;18(6):e0280416. doi: 10.1371/journal.pone.0280416 (PMC10292717; doi:10.1371/journal.pone.0280416)
Supplement: S1 File — (PDF) [file pone.0280416.s001.pdf]

Supportive Information

Bouts of rest and physical activity in C57BL/6J mice

by Karin Pernold, Eric Rullman and Brun Ulfhake.

Fig. 1

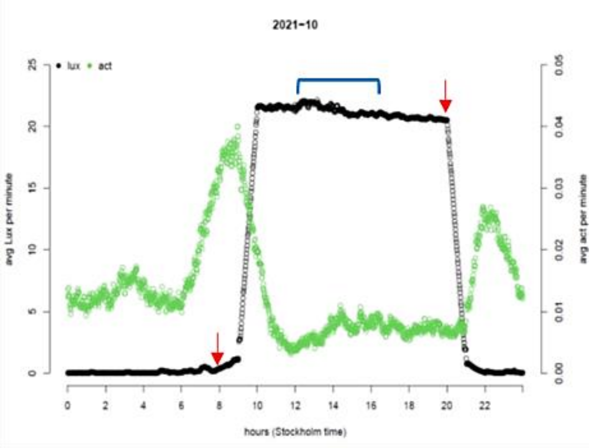

Fig. 1 Panel shows light on (left red arrow in each panel) and lights off (red arrow to the right). Abscissa is local time and left ordinate Lux at the rack front. Right ordinate shows EAD min^{-1} across the LD cycle for Fx3 cohort used in this study. Blue bracket indicates time for cage change and the immediate increase in activity that follows. Note that the response in activity precedes lights on, peaks during the dawn period and decreases towards the end of dawn. Dusk is followed by an increase in EAD, the ramp up occurs during the first hour following dusk initiation. Data on Lux were recorded by the REM unit on the DVC rack, EAD by the DVC.

Fig 2

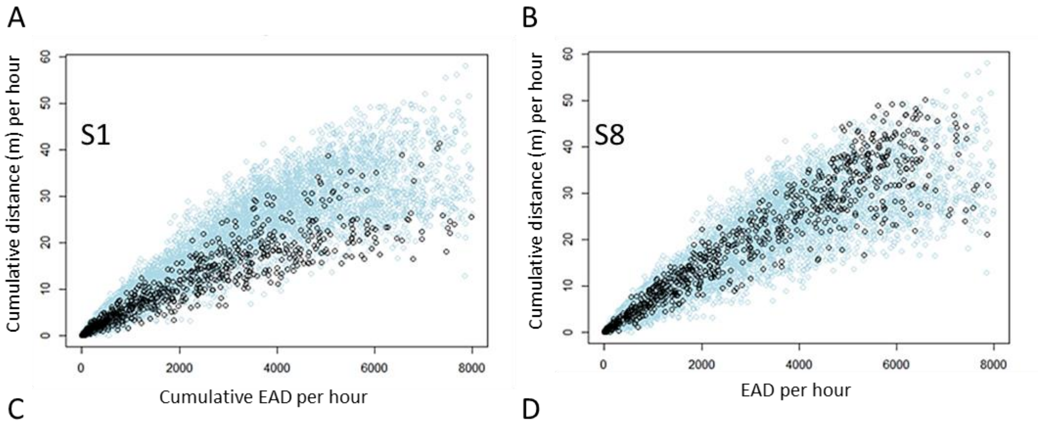

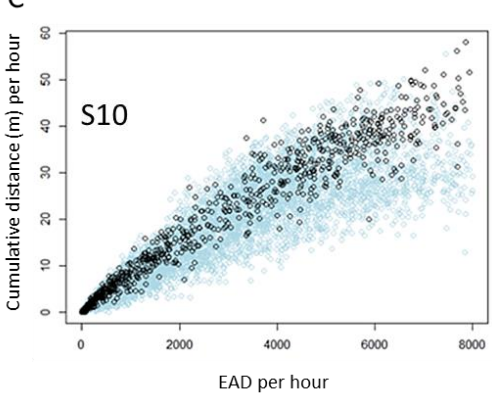

D

Cumulative distance vs EAD h^{-1}			
Animal	S (rank sum)	R_S (rho)	p-value
S1	4.26E+06	0.9741962	< 2.2e-16
S2	6.14E+06	0.962732	< 2.2e-16
S3	3.54E+06	0.979216	< 2.2e-16
S4	3.64E+06	0.9784048	< 2.2e-16
S5	4.94E+06	0.9690034	< 2.2e-16
S6	4.87E+06	0.9709534	< 2.2e-16
S7	4.89E+06	0.9678261	< 2.2e-16
S8	4.55E+06	0.9727109	< 2.2e-16
S9	3.11E+06	0.9817754	< 2.2e-16
S10	2.78E+06	0.9831943	< 2.2e-16

Fig 2 A-C Plots showing the correlation between distance travelled per hour (the tracking metric, ordinate) and number of electrode activations (EAD metric, abscissa) for three of the single-housed (S1, S8 and S10) mice (black circles). Each data set was plotted on the whole data set of 10 mice (green circles). NB: difference between the mice. In D, the Spearman rank correlation data for all 10 mice have been compiled.

Fig 3

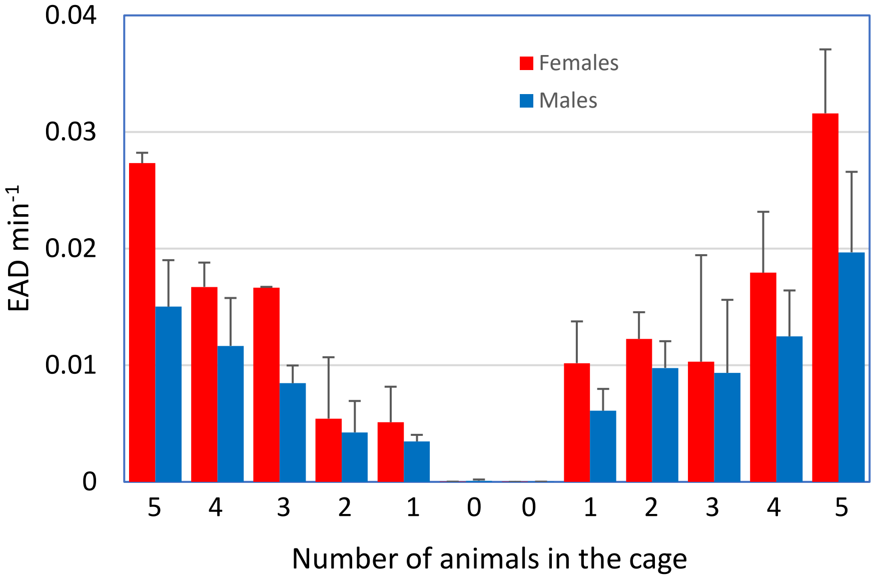

Fig 3 Histogram showing the impact on EAD min^{-1} by stepwise lowering the housing density from 5 to 1 mice (one mouse was removed each consecutive day) and then adding the mice back one by one up to five. Female cages (n=3) in red and male cages (n=5) in blue; average EAD value min-1 across 24 h (λ=2) with standard deviation has been indicated.

Fig 4

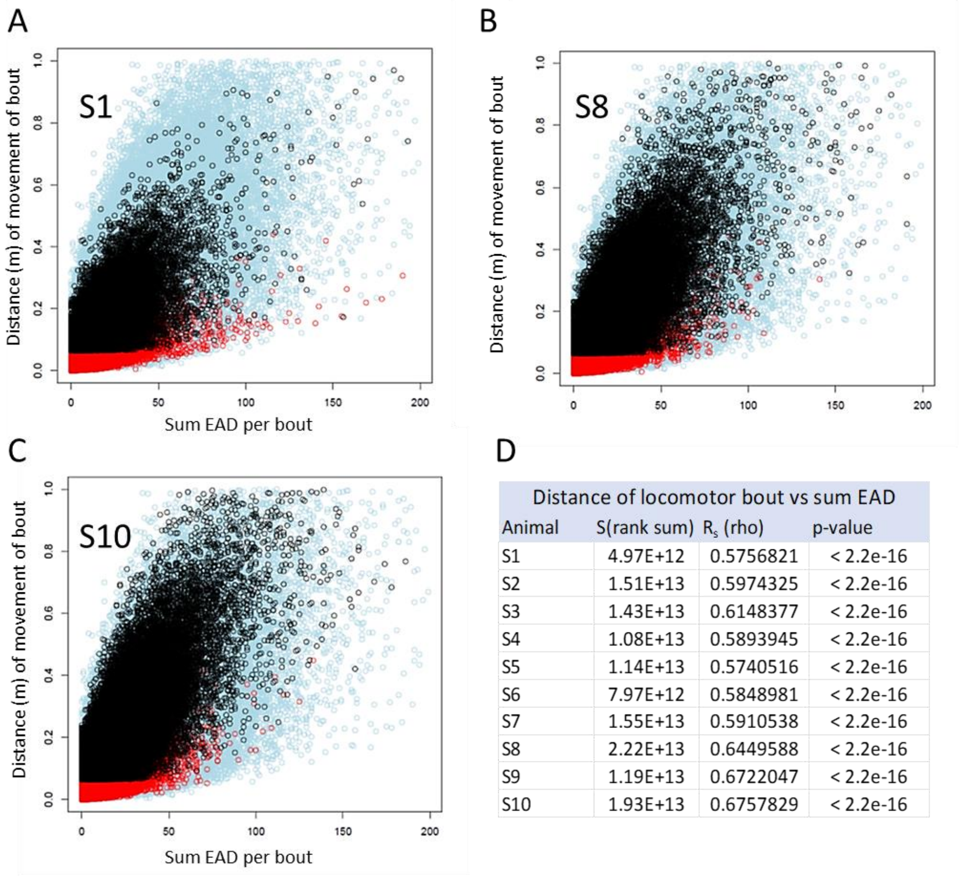

Distance of locomotor bout vs sum EAD			
Animal	S(rank sum)	R_s (rho)	p-value
S1	4.97E+12	0.5756821	< 2.2e-16
S2	1.51E+13	0.5974325	< 2.2e-16
S3	1.43E+13	0.6148377	< 2.2e-16
S4	1.08E+13	0.5893945	< 2.2e-16
S5	1.14E+13	0.5740516	< 2.2e-16
S6	7.97E+12	0.5848981	< 2.2e-16
S7	1.55E+13	0.5910538	< 2.2e-16
S8	2.22E+13	0.6449588	< 2.2e-16
S9	1.19E+13	0.6722047	< 2.2e-16
S10	1.93E+13	0.6757829	< 2.2e-16

Fig 4 A-C Plots showing the correlation between distance travelled per bout (the tracking metric, ordinate) and number of electrode activations per bout (EAD metric, abscissa) for three (S1, S8 and S10) of the single-housed mice (excluding outliers with distance >1m or EAD >200; <1% of the data set). Locomotor bouts have been indicated by black circles and MOTS bouts by red circles. Data for each of the three mice was plotted on the whole data set from 10 mice (green circles). NB: difference between the mice. In D, the Spearman rank correlation data for locomotor bouts vs EAD for all 10 mice have been compiled.

Fig 5

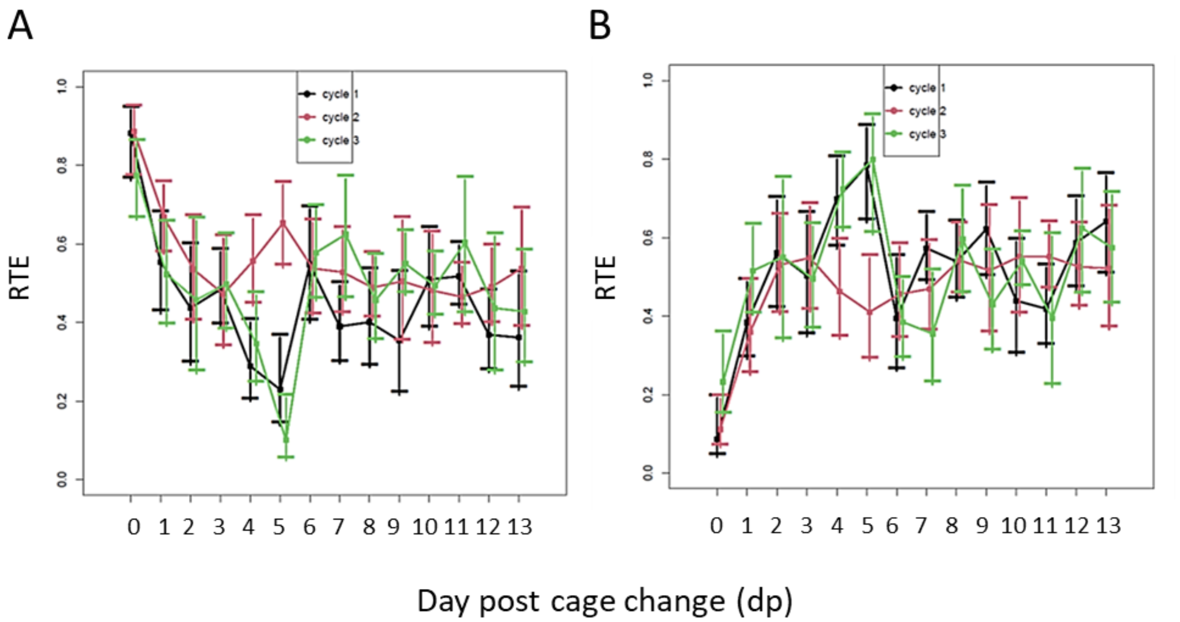

Fig 5 A-B show the relative effect size of days-post-cage-change (dp) and cage-change-cycle (CC, colour coded in black, red 1176 and green), respectively. In A (model: PA ~dp * CC, the impact is by dp (p=6.2E-11) and not by CC (p=0.16). B shows that CC has no significant impact on long rest (p=0.54) while dp has a strong effect (p=4.3E-11).

Fig 6

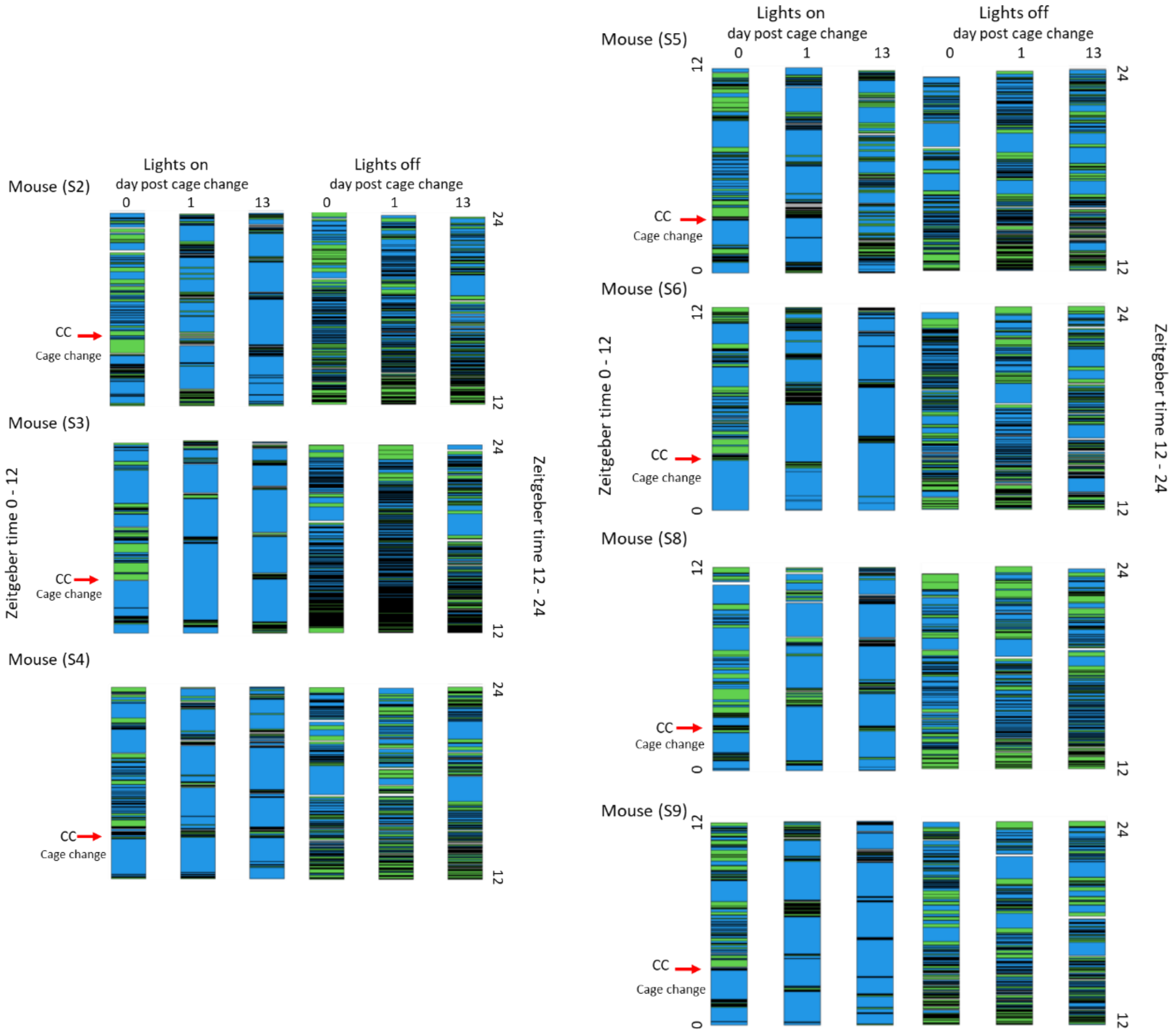

Fig 6 Pattern of long-rest episodes during lights on (ZT 0-12) and lights off (ZT 12-24) (DL 12:12) for the mice S2-S6 and S8-S9 mice housed in isolation. Long rest bouts indicated by blue colour. Bouts disrupting long-rest periods in black/green. Left ordinate is 12h lights on to the left and 12h lights off on the ordinate to the right. Columns are day post cage change (dp) where 0 is the day of the cage change (CC; red arrow indicates the time for CC) for lights on (to the left) and lights off (to the right). Dp1 the day after CC and dp13 the day before the next CC.

Fig. 7

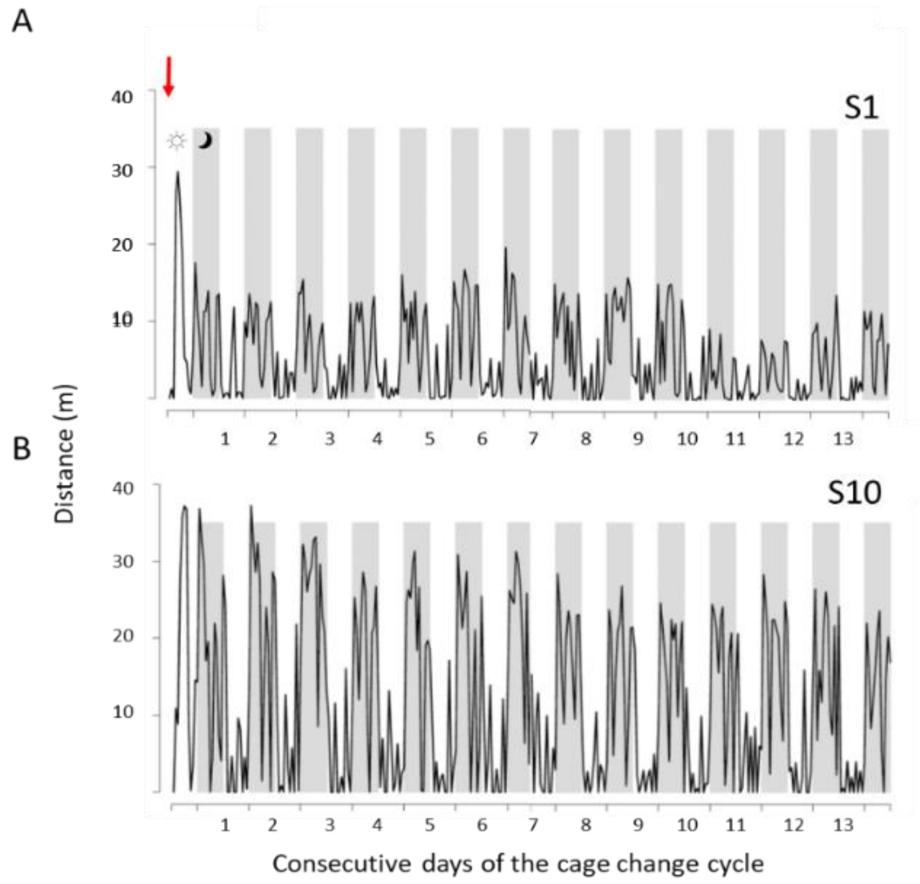

Fig 7 A-B show mouse S1 and S10 distance per hour (m; ordinate) made through the LD cycle of each consecutive day (abscissa) of a cage change cycle. Time of the cage change marked with red arrow in top panel. L (ZT 0-12) on white background, while D (ZT 12-24) have a grey background; see symbols in upper panel.

Fig. S8

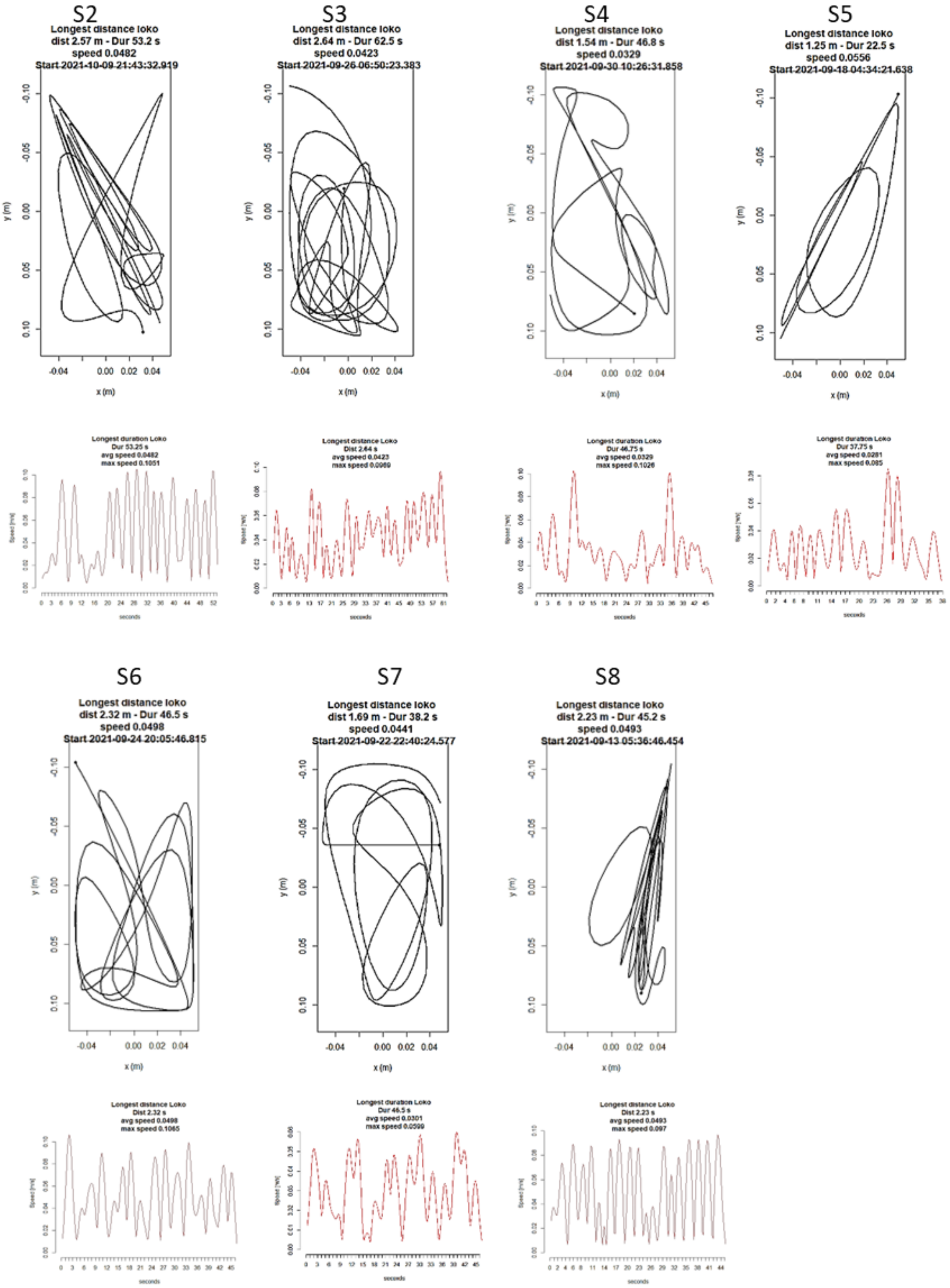

Fig 8 Panels show longest recorded locomotor bout for mouse S2-S8, respectively. Lower panels with red traces show corresponding speedograms. Plots and speedograms by the trajaR software.

Fig 9

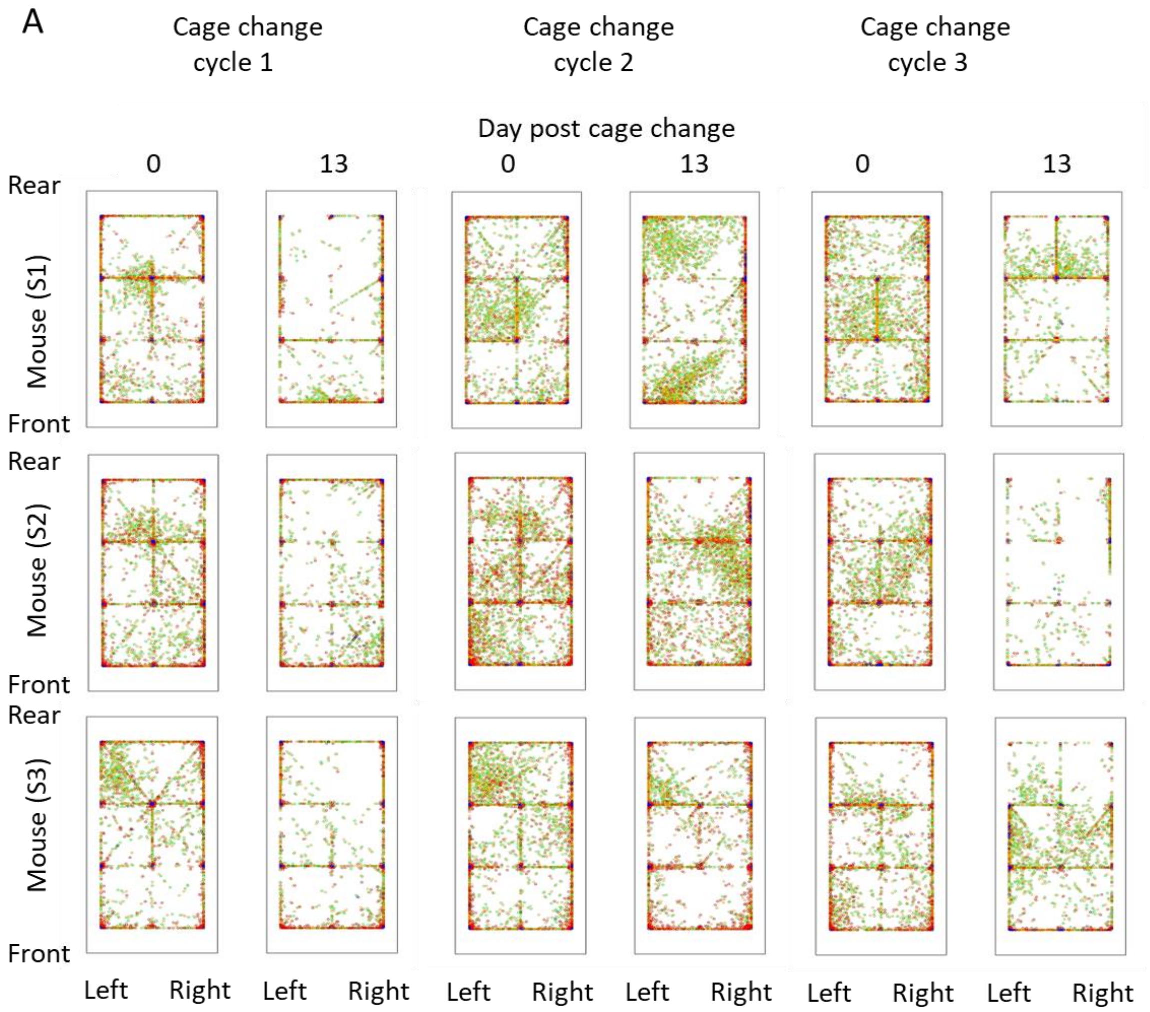
A
Cage change cycle 1
Cage change cycle 2
Cage change cycle 3
Day post cage change
0
13
0
13
0
13
Rear
Mouse (S1)
Front
Rear
Mouse (S2)
Front
Rear
Mouse (S3)
Front
Left
Right
Left
Right
Left
Right
Left
Right
Left
Right
Left
Right

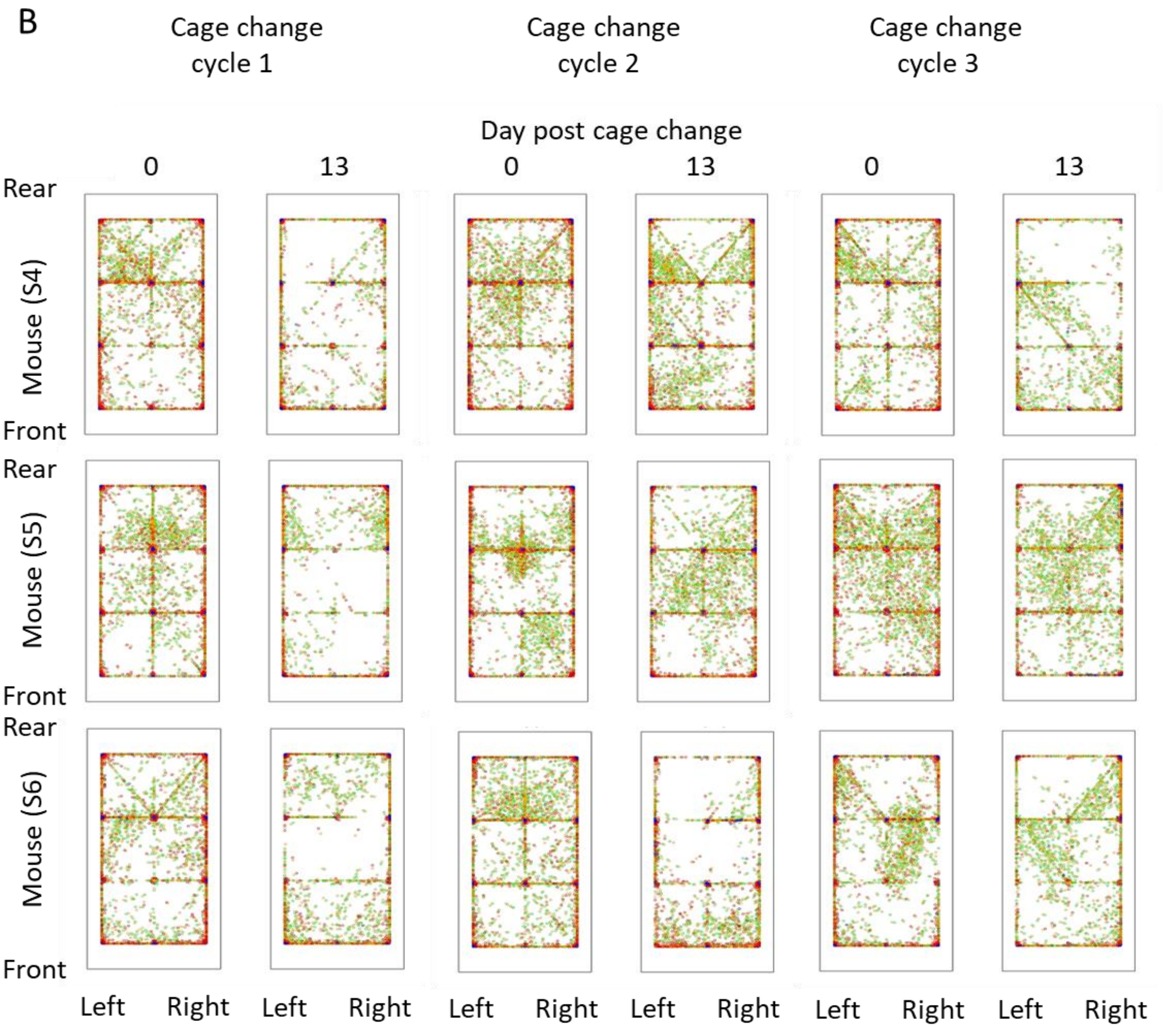
B
Cage change cycle 1
Cage change cycle 2
Cage change cycle 3
Day post cage change
0
13
0
13
0
13
Rear
Mouse (S4)
Front
Rear
Mouse (S5)
Front
Rear
Mouse (S6)
Front
Left
Right
Left
Right
Left
Right
Left
Right
Left
Right
Left
Right

66

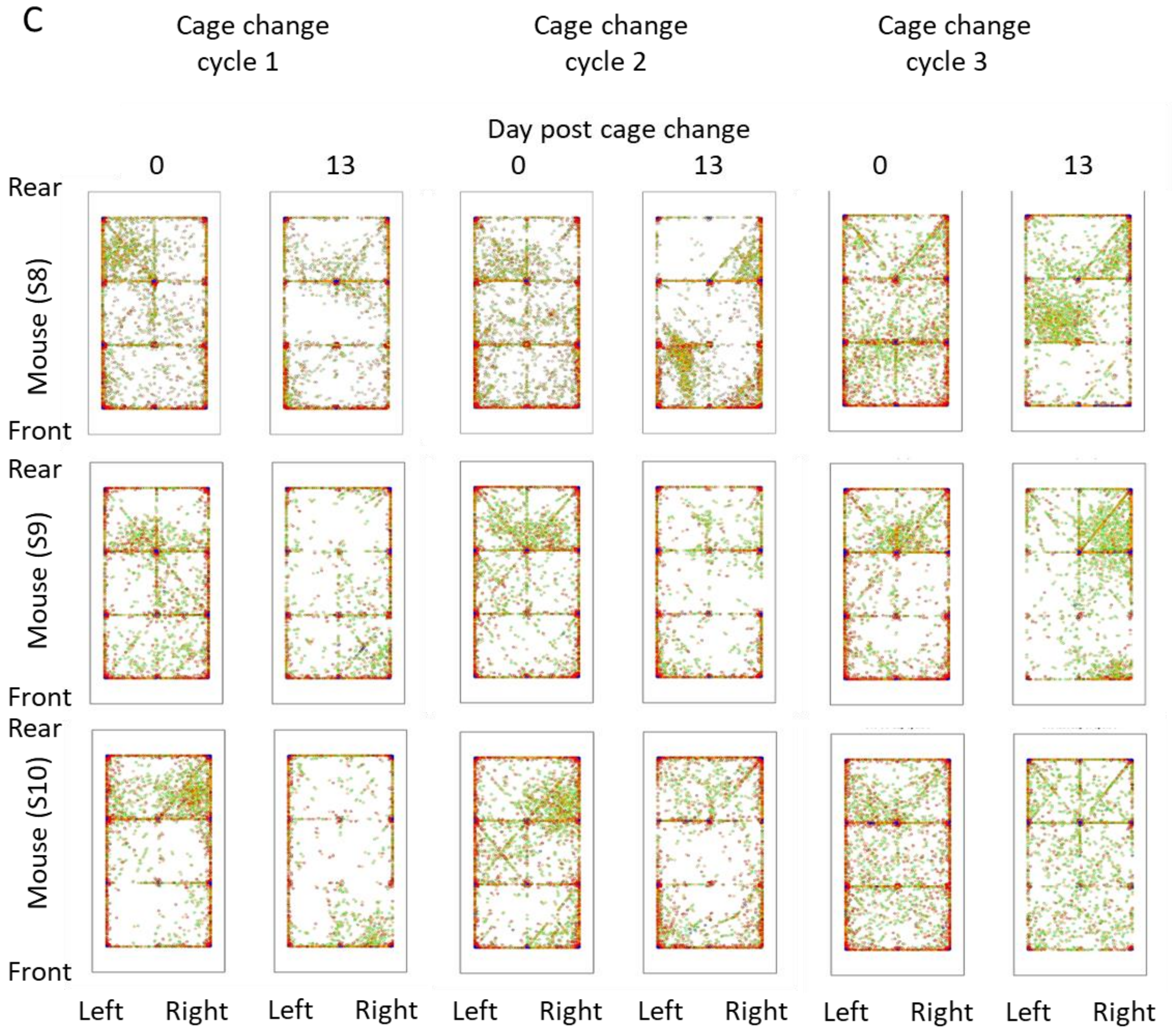

Fig. 9 (A) Densitograms showing the distribution of bout starting coordinates during 24h on dp0 and dp13 in cage change cycles 1 – 3 (columns) for (A) mice S1-S3, (B) mice S4-S6, and (C) mice S8-S10., respectively (rows). Cage front and rear and left (L) and right (R) have been indicated. Key to colour: Blue is long rest bout, orange is short rest bout, green is MOTS, and red is locomotor bout.

Fig. 10

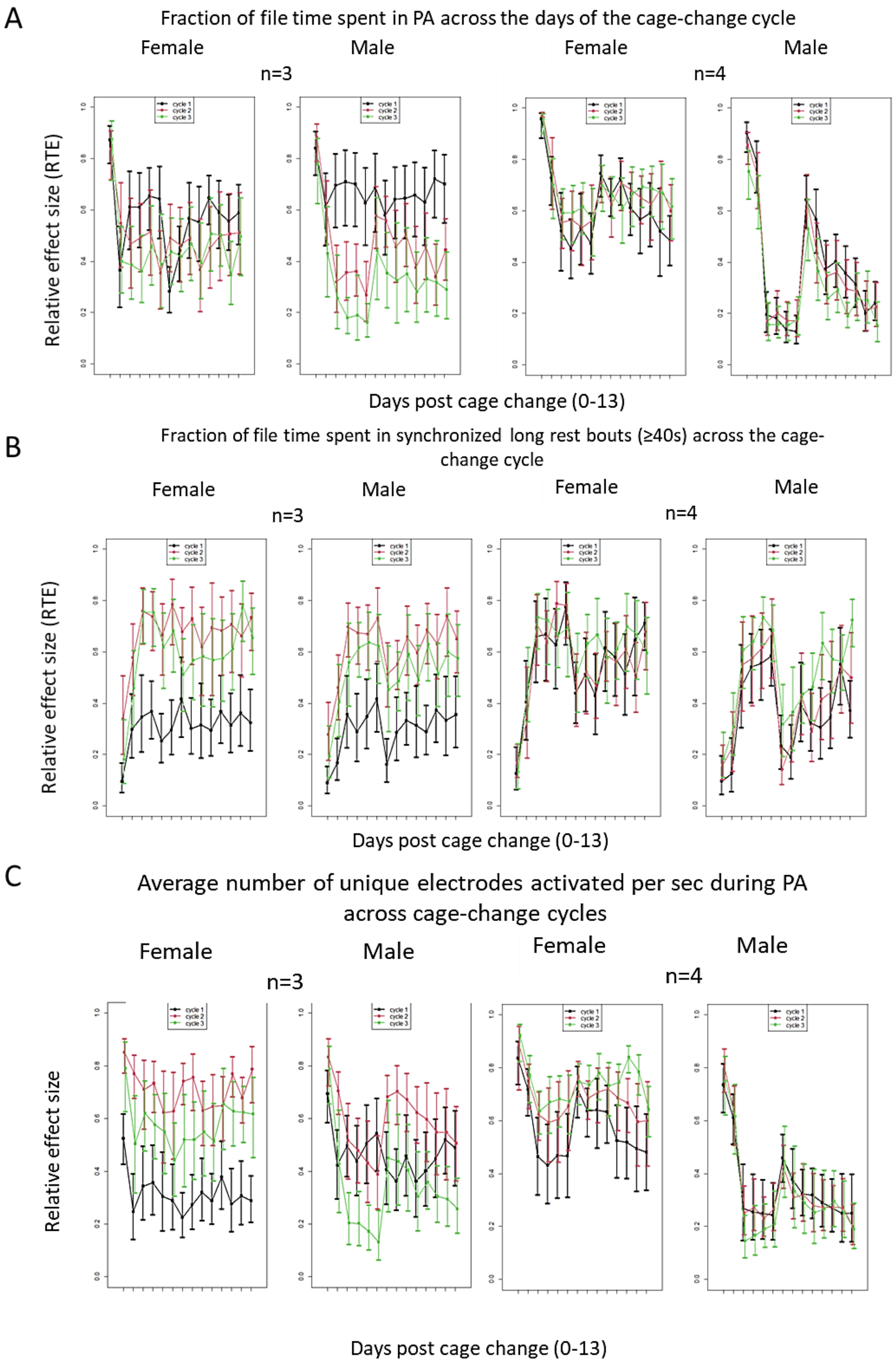
A
Fraction of file time spent in PA across the days of the cage-change cycle
Female
Male
n=3
Female
Male
n=4
Relative effect size (RTE)
Days post cage change (0-13)
B
Fraction of file time spent in synchronized long rest bouts (≥40s) across the cage-change cycle
Female
Male
n=3
Female
Male
n=4
Relative effect size (RTE)
Days post cage change (0-13)
C
Average number of unique electrodes activated per sec during PA across cage-change cycles
Female
Male
n=3
Female
Male
n=4
Relative effect size
Days post cage change (0-13)

Fig. 10 A shows the relative effect size (RTE; ordinate) on PA of sex, dp (0-13; abscissa) and cage change cycle (CC; colour coded black, red, and green in each panel) have been indicated at densities x3 and x4 for female and male mice. B shows the relative effect size (RTE; ordinate) on long rest of sex, dp (0-13; abscissa) and cage change cycle (CC; colour coded black, red, and green in each panel) have been indicated at densities x3 and x4. Dp had the strongest impact on time in long rest, followed by sex at density n=4. C shows the relative effect size (RTE; ordinate) on number of unique electrode activations s^{-1} of sex, dp (0-13; abscissa) and cage-change cycle (CC; colour coded in each panel; black, red, and green) have been indicated.
